# SCM-25: A Zeolite with Ordered Meso-cavities Interconnected by 12 × 12 × 10-Ring Channels Determined by 3D Electron Diffraction

**DOI:** 10.1021/acs.inorgchem.1c03632

**Published:** 2022-01-25

**Authors:** Yi Luo, Wenhua Fu, Bin Wang, Zhiqing Yuan, Junliang Sun, Xiaodong Zou, Weimin Yang

**Affiliations:** ‡ Department of Materials and Environmental Chemistry, Stockholm University, SE-106 91 Stockholm, Sweden; § State Key Laboratory of Green Chemical Engineering and Industrial Catalysis, Sinopec Shanghai Research Institute of Petrochemical Technology, 1658 Pudong Beilu, Shanghai 201208, China; ∥ College of Chemistry and Molecular Engineering, Beijing National Laboratory for Molecular Sciences, Peking University, Beijing 100871, China

## Abstract

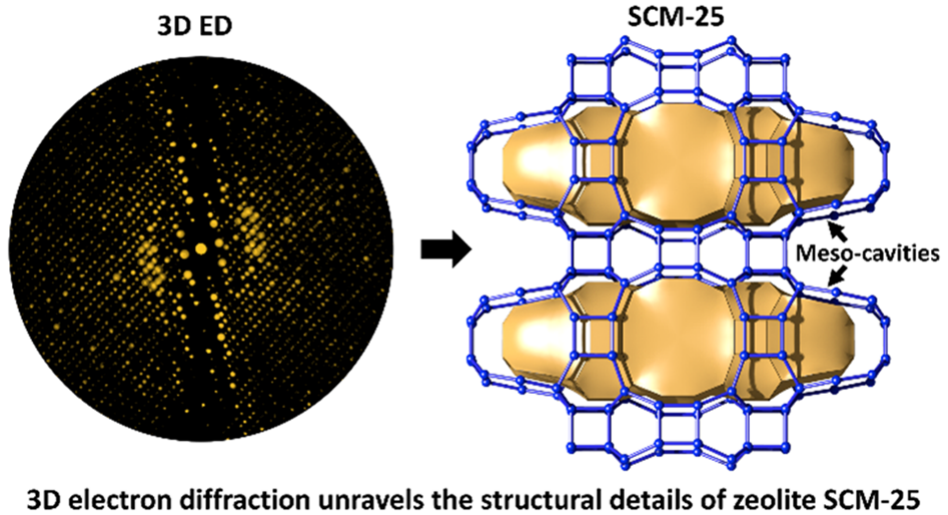

Zeolites with large cavities that are accessible via wide pore windows are desirable but very rare. They have been dominantly used as catalysts in industry. Here we report a novel porous germanosilicate SCM-25, the zeolite structure containing ordered meso-cavities (29.9 × 7.6 × 6.0 Å^3^) interconnected by 10- and 12-ring channels. SCM-25 was synthesized as nanosized crystals by using a simple organic structure-directing agent (OSDA). Three-dimensional (3D) electron diffraction shows that SCM-25 crystallizes in the orthorhombic space group *Cmmm* with *a* = 14.62 Å, *b* = 51.82 Å, *c* = 13.11 Å, which is one of the zeolites with the largest unit cell dimensions. We demonstrate that 3D electron diffraction is a powerful technique for determining the complex structure of SCM-25, including the disorders and distributions of framework atoms silicon and germanium. SCM-25 has a high surface area (510 m^2^/g) and high thermal stability (700 °C). Furthermore, we propose a potential postsynthetic strategy for the preparation of zeolites with ordered meso-cavities by applying the ADOR (assembly–disassembly–organization–reassembly) approach.

## Abstract 2

Porous germanosilicate zeolite SCM-25 was synthesized using a simple, commercially available organic structure-directing agent. 3D electron diffraction (3D ED) revealed that its framework contains ordered meso-cavities interconnected by 12 × 12 × 10-ring channels. SCM-25 is among zeolites with largest unit cell dimensions. Positions of all framework atoms, including disorders and distribution of silicon and germanium, could be accurately determined by 3D ED. A potential postsynthetic strategy for preparing zeolites with ordered meso-cavities is proposed.

## Introduction

Zeolites are technologically important crystalline microporous materials that have wide applications in catalysis, adsorption/separation, and ion-exchange.^1^ Their framework structures are built with TO_4_ tetrahedra (T= Si, Ge, Al, and B, etc.), which connect to each other through vertex-sharing of oxygen atoms in the tetrahedra. Different arrangements and connections of the TO_4_ tetrahedra can form various crystalline structures with tunable chemical compositions and uniform pores and/or cavities of molecular dimensions.^2^ The microscopic structure features of a zeolite, especially the size and shape of pores/cavities and the dimensionality of channels, significantly govern its macroscopic properties such as size and shape-selectivity and molecular diffusion in catalysis and adsorption/separation.^3−5^ The search for zeolites with novel framework topologies and unique pore architectures for improved catalysis and adsorption/separation has always been the object of many researches worldwide.

To date, the International Zeolite Association (IZA) has authorized 255 different zeolite framework structures, which can be classified depending on the ring size of pore openings (defined by the number of tetrahedra forming the ring), the dimensionality of channel systems, or the presence of cavities.^6^ Pores defined by 8, 10, and 12 TO_4_ tetrahedra are considered as small pores (8-ring), medium pores (10-ring), and large pores (12-ring), respectively. Among all available zeolites, those with large cavities have been widely applied in many industrial chemical processes and dominate the synthetic zeolite market.^6−8^ Some examples are **LTA** (8 × 8 × 8-ring, sphere-like cavity of 10.8 Å in the largest dimension), **CHA** (8 × 8 × 8-ring, ellipse-like cavity of 9.9 Å), **MWW** (10 × 10-ring, ellipse-like cavity of 19.0 Å), and **FAU** (12 × 12 × 12-ring, sphere-like cavity of 13.0 Å). This is because their pore architectures allow both molecular diffusion and shape-selective catalysis/adsorption.^7−11^ Therefore, zeolites with large cavities interconnected by well-defined pore windows or channels are of great interest.^5,12−15^ However, studies on these excellent zeolites have shown that their maximum capacities in many applications could still be optimized by increasing the size of pores and/or cavities.^16−18^

Significant efforts have been made to prepare novel zeolites with unique pore structures, especially those containing extra-large pores (>12-ring, >8.0 Å).^19−21^ Currently, the main strategies used for preparing zeolites with novel structures are as follows: (i) predesignation of organic structure-directing agent (OSDA), (ii) heteroatom substitution, and (iii) topotactic conversion of 2D to 3D, 3D to 3D, and 3D to 2D materials, including the assembly–disassembly–organization–reassembly (ADOR) approach.^22−24^ In the past decades, through the strategy of using predesigned large, bulky OSDAs and/or heteroatoms, a series of novel extra-large pore zeolites (EMM-23(*-EWT),^25^ ITQ-33(**ITT**),^26^ ITQ-37(**-ITV**),^27^ ITQ-40(**-IRY**),^28^ ITQ-44(**IRR**),^29^ ITQ-51(**IFO**),^30^ and ITQ-43^31^ etc.) have been synthesized, some with pores beyond 20 Å, extending from the microporous range (<20.0 Å) to mesoporous range (20.0–50.0 Å).^1^ However, large bulky OSDAs are often difficult to prepare and are expensive, which hinders the applications of those extra-large pore zeolites.^22^ The ADOR approach takes advantage of the weakness of the structures of germanosilicate zeolites to achieve the selective dissolution of germanium from the structure. It provides the opportunities to prepare novel zeolite families with continuously controllable porosities that are unfeasible by traditional solvothermal synthetic methods. However, so far, the zeolites afforded by ADOR have either irregular mesopores or micropores with reduced pore sizes compared to the parent zeolite.^24^ Alternatively, mesopores can also be introduced into zeolite crystals via various postsynthetic approaches such as acid, alkaline, and steam treatments.^32^ However, it is challenging to control the size, shape, and location of mesopores by those treatments. The resulting mesopores are mostly irregular in terms of sizes and distributions, which may sacrifice the crystallinity and shape-selectivity of zeolite materials.^11^

Zeolites with large cavities that are accessible via large pore windows are desirable, because they allow large molecules to enter the cavities and access the catalytic centers and can also enhance the shape selectivity and molecular diffusion. While the synthesis of zeolites with large/meso-cavities connected to small 8-ring pore windows has been realized in the ABC-6 family,^13,33^ there is a lack of strategy to synthesize zeolites with ordered meso-cavities accessible via medium (10-ring) and large (12-ring) pore windows. Herein, we report a germanosilicate SCM-25, a zeolite with a hierarchical pore architecture built of meso-cavities fully interconnected by medium and large pores. SCM-25 was first synthesized using a simple and commercially available OSDA in 2019.^34^ The structure of SCM-25 was determined from nanosized crystals by 3D electron diffraction (3D ED),^35−39^ more specifically continuous rotation electron diffraction (cRED).^40^ The framework of SCM-25 is isostructural to that of recently reported HMP-16^41^ and closely related to that of ITQ-21,^42^ which has an intersecting 3D 12 × 12 × 12-ring channel system. A potential postsynthesis strategy for preparing zeolites with meso-cavities is proposed based on the structural relationship between SCM-25 and ITQ-21.

## Results and Discussion

The germanosilicate zeolite SCM-25 was synthesized as nanosized platelike crystals (∼700 × 500 × 30 nm^3^, Figure S1) in fluoride medium using 1,1,3,5-tetramethyl piperidinium hydroxide (1,1,3,5-TMPOH) as an organic structure-directing agent (OSDA).^34^ It crystallized from a gel with a molar composition of 0.5 1,1,3,5-TMPOH/0.667 SiO_2_/0.333 GeO_2_/0.5 HF/7 H_2_O at 175 °C for 14 days under dynamic conditions. The detailed descriptions of the synthesis conditions are given in the Supporting Information.

The ^13^C solid-state MAS NMR of as-made SCM-25 indicates that the OSDAs are intact and accommodated in the structure (Figure S2). The Si/Ge molar ratio of the solid sample is 2.4, as revealed by the inductively coupled plasma-atomic emission spectrometry (ICP-AES) analysis (Table S1). In situ PXRD shows that SCM-25 has high thermal stability (>700 °C, Figure S3). Its framework structure was retained after removing the OSDAs by calcination in air at 550 °C (Figure S5). The ^29^Si solid-state MAS and ^1^H to ^29^Si CPMAS NMR spectra of the calcined SCM-25 show that most Si atoms are connected to four TO_4_ (Q4 species, Si(OT_4_), T = Si, Ge, Figure S6).^28,43^ The ^19^F solid-state MAS NMR spectrum implies the existence of double 4-ring (*d*4*r*) units in the structure (Figure S6).^44^ N_2_ adsorption measurements of SCM-25 show a Brunauer–Emmett–Teller (BET) surface area of 510 m^2^/g with a micropore volume of 0.18 cm^3^/g (Figure S7). Meanwhile, a bimodal pore size distribution centered at 6.9 and 8.2 Å was observed (Figure S7).

**Figure 1:**
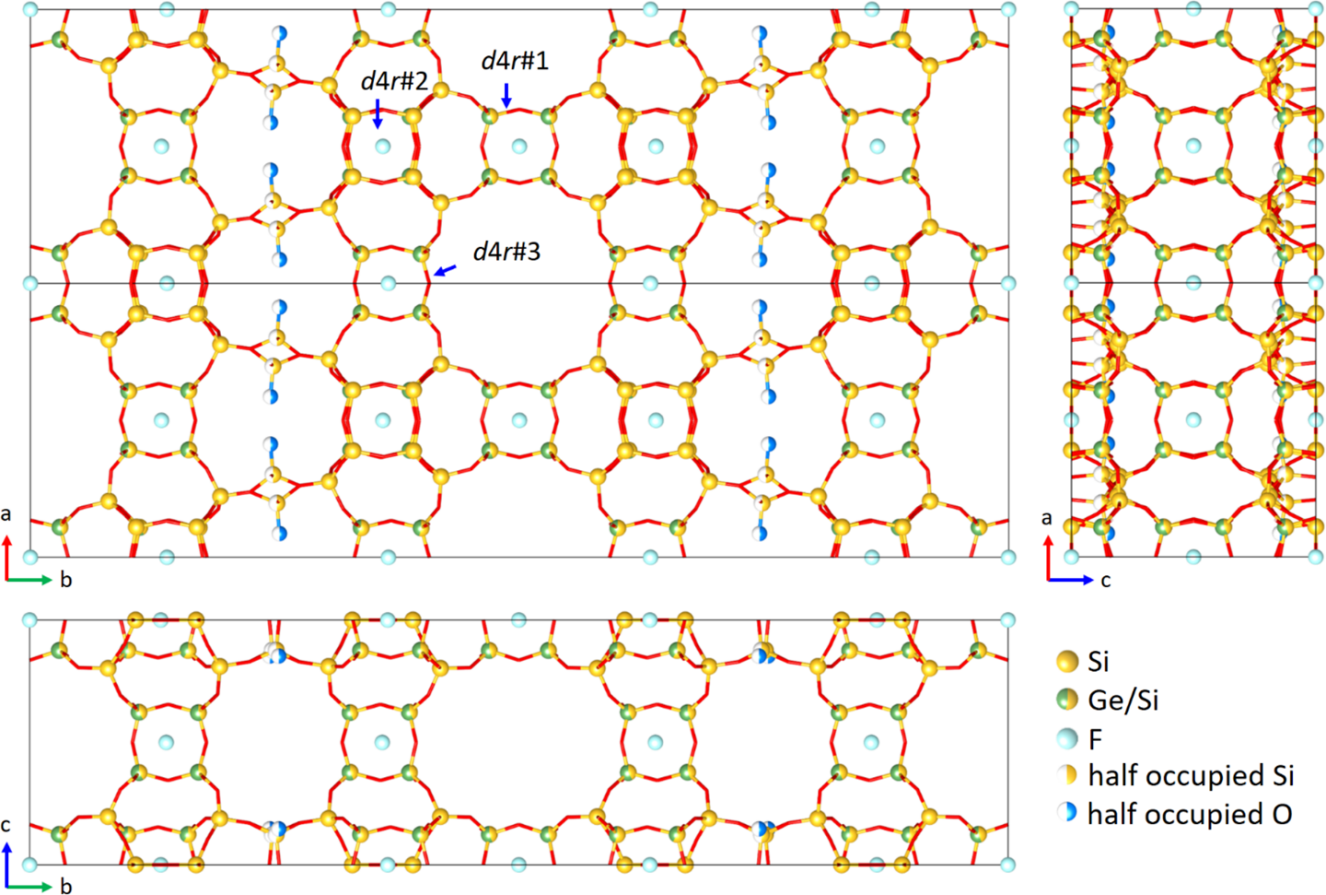
Projections of the framework structure of as-made SCM-25 (2 × 1 × 1 unit cells) determined from the cRED data. The occupancies of Ge atoms were refined, and the locations of F^–^ ions were accurately identified. The half-occupied Si and O atoms show the disorder. The terminal O atoms are in blue, highlighting the disorder.

**Figure 2:**
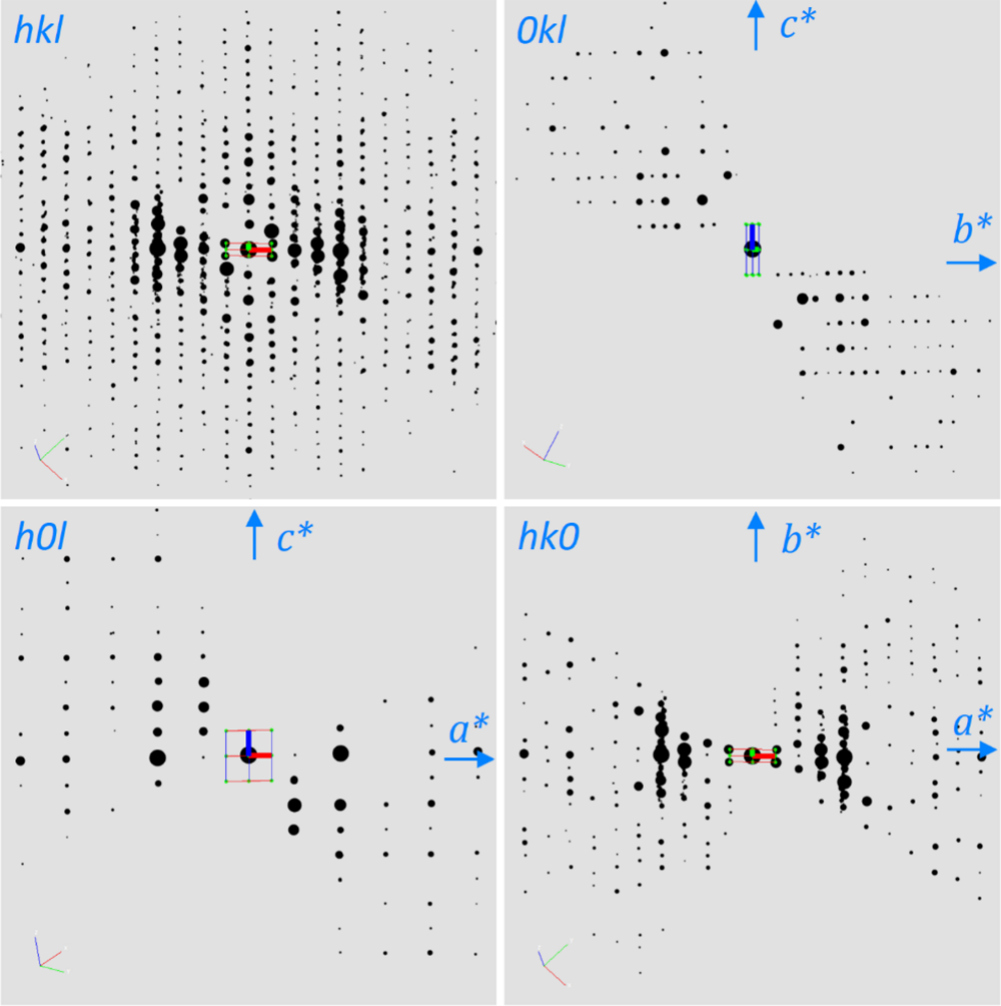
3D reciprocal lattice of as-made SCM-25 reconstructed from the cRED data. The reflection conditions deduced from the 3D reciprocal lattice and the 2D slices of 0*kl*, *h*0*l*, and *hk*0 are *hkl*: *h* + *k* = 2*n*, 0*kl*: *k* = 2*n*, *h*0*l*: *h* = 2*n*, *hk*0: *h* + *k* = 2*n*, and *h*00: *h* = 2*n*. The possible space groups are *Cmmm*, *Cmm*2, or *C*222.

Because of the nanosize of SCM-25 crystals that are too small for single-crystal X-ray diffraction, continuous rotation electron diffraction (cRED) was used for structure determination of SCM-25 (Figures 1 and 2). cRED data were collected on a JEOL JEM2100 transmission electron microscope (TEM) at 200 kV equipped with a Timepix hybrid pixel detector using the program *Instamatic* developed in our lab.^40^ A cRED data set with 342 diffraction frames and rotation range of 90.61° was collected within 3.4 min (Table S2), which shows that SCM-25 has a large *C*-centered orthorhombic cell of *a* = 14.62 Å, *b* = 51.82 Å, *c* = 13.11 Å, as identified using the program *XDS*.^45^ Based on the reflection conditions observed from the 3D reciprocal lattice reconstructed from the cRED data by the program *REDp*(46) (Figure 2), three possible space groups, *Cmmm*, *Cmm*2, and *C*222, were deduced. Integrated intensities of the reflections were obtained from the cRED data using *XDS* with a high resolution (0.80 Å) and completeness (89.8%) (Table S2). The framework structure of SCM-25 could be solved with the space group *Cmmm* using the program *SHELXT*.^47^ All the 10 framework T atoms and 24 of 26 framework oxygen atoms in the asymmetric unit were located directly (Figures 1 and S8). While 9 of the 10 T atoms are four-connected, one is three-connected and disordered at two symmetry-related positions. Two terminal O atoms coordinated to the disordered T atom could not be located by *SHELXT* due to the low occupancy (0.5). They were added based on the geometry of zeolites.^48^

The structural model of SCM-25 was refined against the cRED data using the program *SHELXLE*.^49^ The highest possible space group *Cmmm* was applied in the refinement, and the more accurate unit cell parameters obtained from the synchrotron PXRD data were used (Table S3). While all T atoms were refined as mixed Si/Ge sites during the initial refinement, the refinement showed that Ge atoms were mainly distributed in the double 4-ring (*d*4*r*) units and the other T-sites were then assigned as Si-site. Restraints on the Si–O bond distances and angles were applied to the disordered terminal Si and O atoms. All atoms were refined anisotropically, and the refinement converged to *R*1 = 0.1848, *wR*2 = 0.4388, and S = 1.684 for 2283 reflections with *F* > 2σ(F) and 286 parameters (Figures 1 and S9, Table S3). The occupancies of Ge atoms and locations of F^–^ ions could be refined without applying any restraints. The Si and Ge compositions of three symmetry-independent *d*4*r* units were refined to be Si_3.4_Ge_4.6_ (*d*4*r*#1), Si_3.5_Ge_4.5_ (*d*4*r*#2), and Si_4.6_Ge_3.4_(*d*4*r*#3) (Figure 1), which are highly consistent with the results revealed by the ^19^F NMR spectrum (Figure S6) and ICP (Table S1). The refined chemical composition is |F^–^_10_|[Si_94.7_Ge_41.3_O_276_] (OSDA and H_2_O molecules were not included in the refinement) and is in good agreement with that (|(C_9_NH_20_F)_10.6_(H_2_O)_7.8_|[Si_96.0_Ge_40.0_O_276_]) calculated from the ICP and TGA analysis (Table S1). The bond lengths are 1.58–1.62 Å (average 1.60 Å) for Si–O and 1.60–1.69 Å (average 1.65 Å) for Si/Ge–O. The T–O–T angles range between 103.3° and 114.9° (average 109.4°), which are chemically reasonable (Table S4). These show the framework structure determined from cRED data is highly reliable. The high *R*-values are not due to the inaccurate structural model but most likely result from dynamical effects due to multiple scattering of electrons in the crystal and the absence of OSDAs in the model.

**Figure 3:**
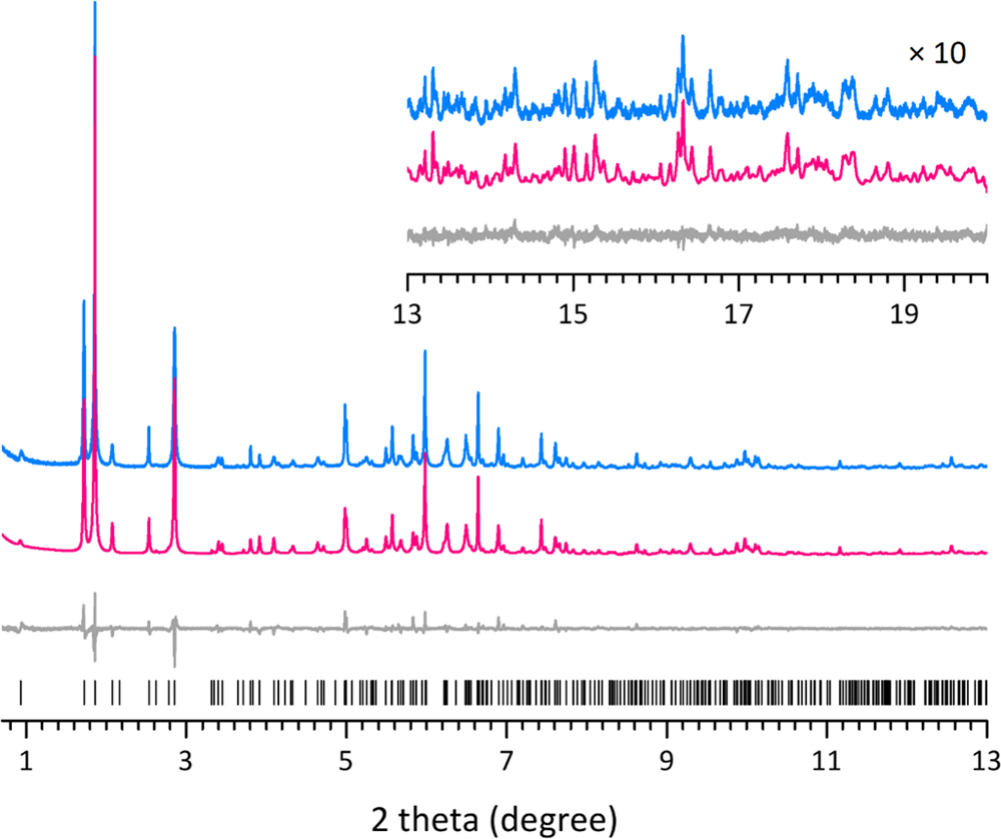
Synchrotron PXRD pattern (blue line, λ = 0.412836 Å) of as-made SCM-25 and its Rietveld refinement. The calculated (pink line) and difference profiles (gray line) of the refinement are also presented. The profiles in the inset have been magnified by 10 times to show more details in the high-angle region. The black tick marks under the profiles are the positions of the Bragg reflections.

Rietveld refinement against the synchrotron powder X-ray diffraction (PXRD) data of as-made SCM-25 was conducted to locate OSDAs in the pores (Figure 3). The framework structure determined by cRED was served as a starting model for Rietveld refinement. During the refinement, the OSDA TMP cations were treated as rigid bodies and located using the simulated-annealing algorithm implemented in *TOPAS V*6.0.^50–52^ All framework atoms were refined with soft geometric restraints of bond distances and angles. The refinement converged with agreement residuals of *R*_B_ = 0.033, *R*_wp_ = 0.170, with *R*_exp_ = 0.099 (Figure 3 and Table S5). The refined chemical composition is |(C_9_H_20_N^+^)(F^–^)(H_2_O)_0.93_|_10_[Si_96.0_Ge_40.0_O_272_]. The framework structure refined against the PXRD data matches very well with the one refined against the cRED data, with an average deviation in atomic positions by 0.04(2) Å for the T atoms and 0.10(4) Å for the O atoms (Tables S4 and S6). There are 10 TMP^+^ cations in each unit cell, which are distributed at four symmetry-independent sites (OSDA#a, #b, #c #d) with a partial occupancy of 0.158, 0.148, 0.174, and 0.146, respectively (Figure S10).^50,51^ Their positive charges are balanced by the 10 F^–^ ions in the *d*4*r* units. These results are consistent with those from NMR, ICP, TGA, etc. Each TMP^+^ cation has several possible locations because of the high symmetry in *Cmmm*. Electron densities from the OSDAs could also be identified from the difference electron density map (Figure S10), which are found both within and at the pore window of the meso-cavities. Figure S11 illustrates the location of the TMP^+^ cations in the meso-cavity.

**Figure 4:**
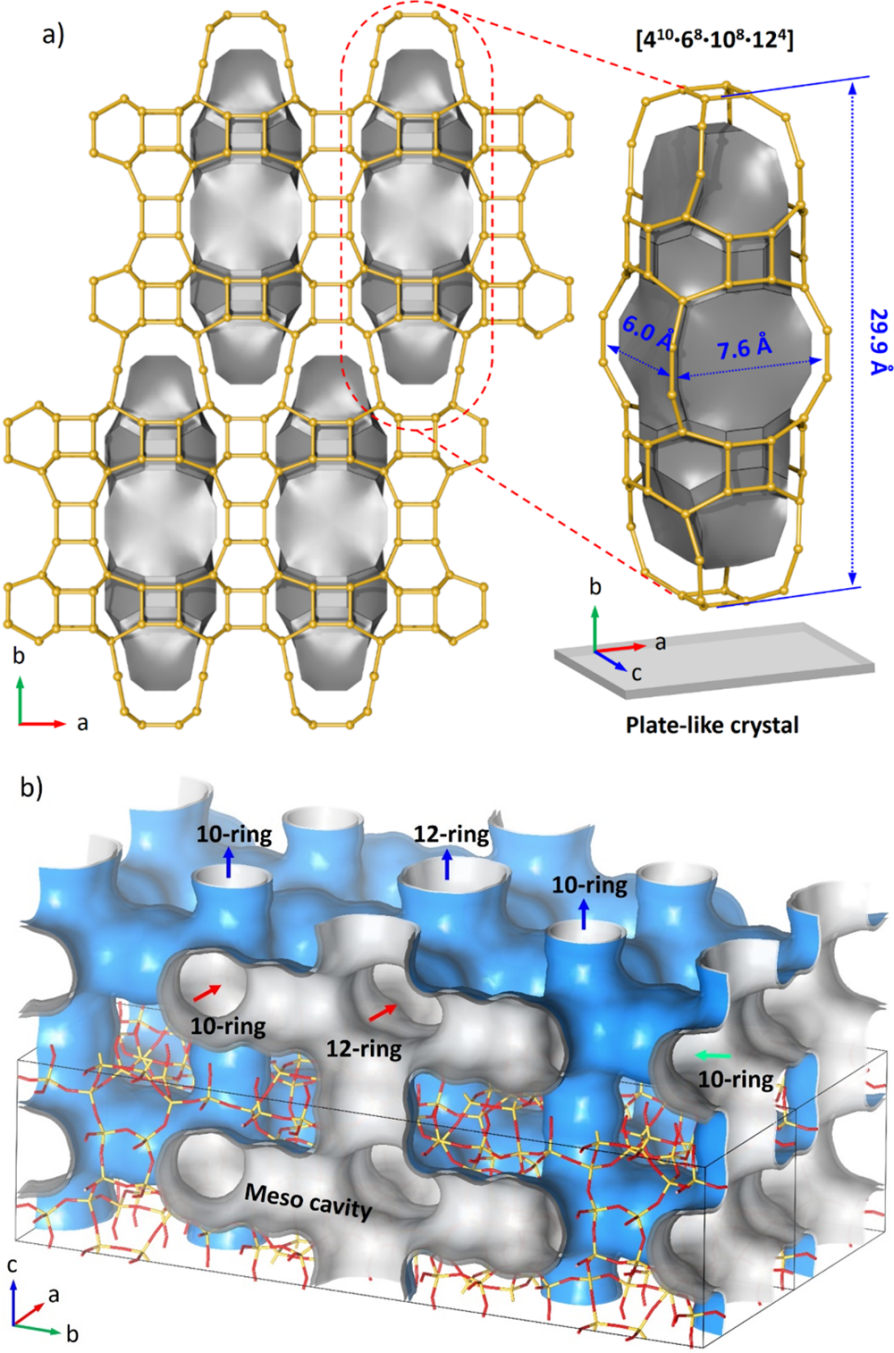
(a) Nature tile of the meso-cavity and its locations in the SCM-25 framework. The largest dimension of the meso-cavity is along the *b*-axis and perpendicular to the crystal. (b) Unique channel system of SCM-25. The envelope is only meant to show the arrangement of the channels and meso-cavities within the framework structure. The image was generated using Material Studio 7.0.^54^

The framework structure of SCM-25 exhibits a novel topology as elucidated by the crystallographic programs *Topos* and 3*dt*.^53^ The three-dimensional network is composed of nine different tiles (Figure S12). The large complex tile with a face symbol [4^10^·6^8^·10^8^·12^4^] represents the novel shuttle-like meso-cavity in SCM-25, with a size of 29.9 × 7.6 × 6.0 Å^3^ (Figures 4a and S12). The shuttle-like meso-cavities are accessible via four 12-ring windows and eight 10-ring windows: two 12-ring (7.6 × 6.8 Å) and four 10-ring (5.4 × 4.7 Å) windows perpendicular to the *c*-axis, and two 12-ring (8.0 × 6.1 Å) and four 10-ring (5.6 × 5.1 Å) windows perpendicular to the *b*-axis (Figure 4b and Figure S13). This gives rise to a very open zeolite structure (framework density: 14.7 T/1000 Å^3^) with a unique 3D 12 × 12 × 10-ring channel system built of the ordered meso-cavities interconnected by the medium and large pores. In addition, we found by electron diffraction that the longest direction of the meso-cavity (along the *b*-axis) is perpendicular to the nanoplates of SCM-25 crystals (Figure S15). These may be beneficial for diffusion, adsorption, and reactivity. The meso-cavities in SCM-25 comprise the unique features of cavities in both **FAU** (12 × 12 × 12-ring) and **MWW** (10 × 10-ring) (Figure S14). All these make SCM-25 promising for catalytic and adsorptive applications.

**Figure 5:**
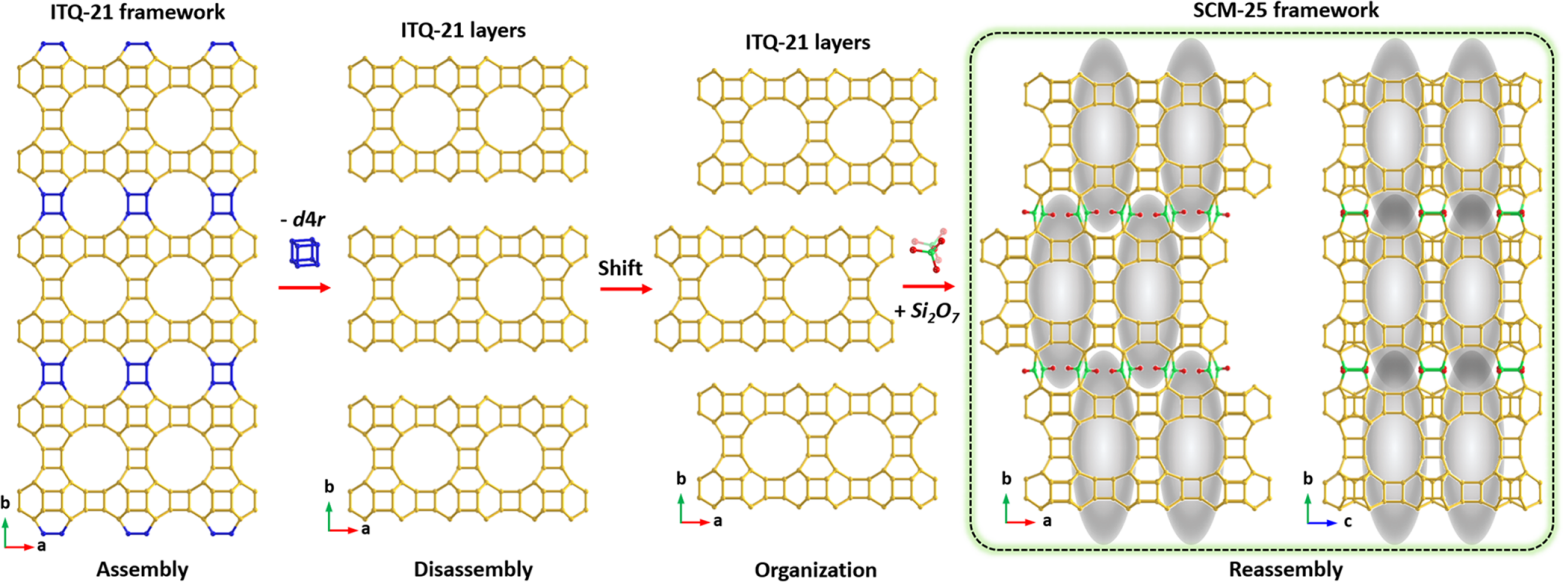
Structural relationship between ITQ-21 and SCM-25. The framework structures of these two zeolites share the same porous layers. The framework structure of ITQ-21 is possible to be transformed into the framework structure of SCM-25 via the ADOR approach. This case inspired us to propose a promising approach to prepare zeolites with meso-cavities in their framework structures. O atoms have been omitted for clarity. The corresponding projections viewed along the *a*-axis are presented in Figure S16.

The framework structure of SCM-25 is closely related to that of zeolite ITQ-21 (space group *Fm*–3*c*, *a* = 27.698 Å),^42^ and both zeolites are built from the same building layer (referred to as ITQ-21 layer) that contains intersecting 12-ring channels along three perpendicular directions (Figure 5). In ITQ-21, the ITQ-21 layers are connected via *d*4*r* units to form a 3D framework with the straight 12-ring channels along the *a*-, *b*-, and *c*-axes. In SCM-25, the neighboring ITQ-21 layers are shifted by 1/2*a* (ca. 7.1 Å) from each other and connected via a tetrahedral pair −O(SiO_2_)–O–(SiO_2_)O–(Si_2_O_7_). The shift of neighboring ITQ-21 layers blocks the straight 12-ring channels perpendicular to the layer and turns the channels into meso-cavities. From the structural point of view, the framework structure of SCM-25 could be constructed from ITQ-21 via the ADOR approach,^24^ as illustrated in Figure 5. The assembled ITQ-21 framework is disassembled into ITQ-21 layers by selective removal of every second *d*4*r* unit along the *b*-axis. The ITQ-21 layers are then reorganized by a shift (1/2*a*) along the *a*-axis (organization) and connected through tetrahedral pairs (Si_2_O_7_) to form the framework structure of SCM-25 (reassembly). We envisage that the ADOR approach could be used to prepare zeolites with ordered meso-cavities based on the same principle as shown in Figure 5. Kasneryk et al. reported that the unfeasible zeolite IPC-12 could be prepared via the ADOR approach from the parent zeolite **UOV** which consists of porous layers.^55^ The *d*4*r* units in the **UOV** framework could be selectively removed to form layers with tunable thickness by tailoring their chemical compositions.^56^ The layers can be rearranged with a shift to interrupt the straight channels and subsequently reassembled by modifying the ADOR conditions.^57^ We believe the ADOR approach has also a potential for synthesizing novel zeolites with ordered meso-cavities by simply blocking straight channels in a known zeolite.

**Figure 6:**
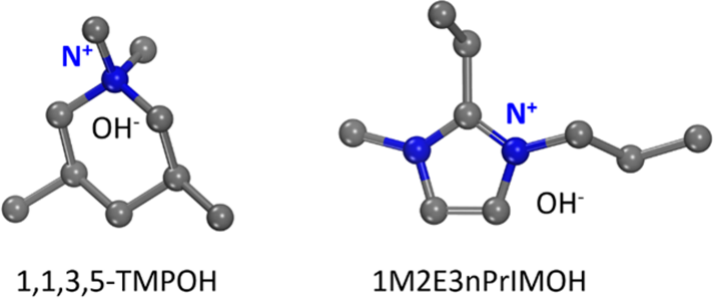
Molecular structures of 1,1,3,5-TMPOH and 1M2E3nPrIMOH, used as the OSDAs for the synthesis of SCM-25 and HPM-16, respectively.

It is worth mentioning that SCM-25 is isostructural to that of recently reported HPM-16.^41^ While SCM-25 was synthesized using a simple and commercially available 1,1,3,5-TMPOH as the OSDA, the OSDA used for the synthesis of HPM-16, 1-methyl-2-ethyl-3-*n*-propylimidazolium hydroxide (1M2E3nPrIMOH), is more complex (Figure 6) and requires several steps to produce.^41^ While the 3D ED data could only provide an initial model of HPM-16 without considering the distribution of silicon and germanium in the framework, we show that accurate framework structure of SCM-25 including the locations of silicon and germanium atoms could be determined. Meanwhile, by Rietveld refinement against the synchrotron PXRD data, we revealed four symmetry-independent sites of TMP^+^ cations in SCM-25, which is different from the two symmetry-independent sites of 1M2E3nPrIM^+^ cations in HPM-16. A comparison of the locations of the OSDAs in the two structures is given in Figure S17. Furthermore, we analyzed the structural relationship between ITQ-21 and SCM-25 and proposed a potential approach to prepare zeolite structures with ordered meso-cavities.

## Conclusions

In summary, we have demonstrated the synthesis and structure determination of a germanosilicate SCM-25, the zeolite containing ordered meso-cavities. SCM-25 was synthesized using a simple and commercially available OSDA and is stable after removing the OSDAs by calcination in air. Recent advances of 3D ED, especially continuous rotation electron diffraction (cRED), made it possible to determine the complex structure of nanosized SCM-25 crystals with high reliability. These are also confirmed by synchrotron PXRD, NMR, ICP, etc. SCM-25 has a unique 3D pore architecture with meso-cavities accessible via large 12-ring and medium 10-ring microporous channels. The high thermal stability together with the unique 3D hierarchical pore architecture makes SCM-25 promising for catalytic and adsorptive applications. In addition, inspired by the structural relationship between SCM-25 and zeolite ITQ-21, we proposed a potential postsynthesis strategy for the preparation of zeolites with ordered meso-cavities.

## Supporting Information Available

Synthetic procedures, characterization methods and data (3D ED, XRD, SEM, TGA, NMR, etc.), and structure refinement details of SCM-25 (PDF)

## Accession Codes

CCDC 2101715–2101716 contain the supplementary crystallographic data for this paper. These data can be obtained free of charge via www.ccdc.cam.ac.uk/data_request/cif, or by emailing data_request@ccdc.cam.ac.uk, or by contacting The Cambridge Crystallographic Data Centre, 12 Union Road, Cambridge CB2 1EZ, UK; fax: +44 1223 336033.

## References

[ref1] Davis M. E. Ordered Porous Materials for Emerging Applications. Nature 2002, 417 (6891), 813–821. 10.1038/nature00785. 12075343

[ref2] Baerlocher C.; McCusker L. B.; Olson D. H. Atlas of Zeolite Framework Types, 6th rev. ed.; Elsevier: Amsterdam, 2007.

[ref3] Smit B.; Maesen T. L. M. Molecular Simulations of Zeolites: Adsorption, Diffusion, and Shape Selectivity. Chem. Rev. 2008, 108 (10), 4125–4184. 10.1021/cr8002642. 18817356

[ref4] Smit B.; Maesen T. L. M. Towards a Molecular Understanding of Shape Selectivity. Nature 2008, 451 (7179), 671–678. 10.1038/nature06552. 18256663

[ref5] Bereciartua P. J.; Cantín Á.; Corma A.; Jordá J. L.; Palomino M.; Rey F.; Valencia S.; Corcoran E. W.; Kortunov P.; Ravikovitch P. I.; Burton A.; Yoon C.; Wang Y.; Paur C.; Guzman J.; Bishop A. R.; Casty G. L. Control of Zeolite Framework Flexibility and Pore Topology for Separation of Ethane and Ethylene. Science 2017, 358 (6366), 1068–1071. 10.1126/science.aao0092. 29170235

[ref6] Baerlocher C.; McCusker L. B. Database of Zeolite Structures. http://europe.iza-structure.org/IZA-SC.

[ref7] Zones S. I. Translating New Materials Discoveries in Zeolite Research to Commercial Manufacture. Microporous Mesoporous Mater. 2011, 144 (1), 1–8. 10.1016/j.micromeso.2011.03.039.

[ref8] Schmidt J. E.; Chen C.-Y.; Brand S. K.; Zones S. I.; Davis M. E. Facile Synthesis, Characterization, and Catalytic Behavior of a Large-Pore Zeolite with the IWV Framework. Chem.—Eur. J. 2016, 22 (12), 4022–4029. 10.1002/chem.201504717. 26833857

[ref9] Vermeiren W.; Gilson J.-P. Impact of Zeolites on the Petroleum and Petrochemical Industry. Top Catal. 2009, 52 (9), 1131–1161. 10.1007/s11244-009-9271-8.

[ref10] Dusselier M.; Van Wouwe P.; Dewaele A.; Jacobs P. A.; Sels B. F. Shape-Selective Zeolite Catalysis for Bioplastics Production. Science 2015, 349 (6243), 78–80. 10.1126/science.aaa7169. 26138977

[ref11] Davis M. E. Zeolites from a Materials Chemistry Perspective. Chem. Mater. 2014, 26 (1), 239–245. 10.1021/cm401914u.

[ref12] Smeets S.; Xie D.; McCusker L. B.; Baerlocher C.; Zones S. I.; Thompson J. A.; Lacheen H. S.; Huang H.-M. SSZ-45: A High-Silica Zeolite with Small Pore Openings, Large Cavities, and Unusual Adsorption Properties. Chem. Mater. 2014, 26 (13), 3909–3913. 10.1021/cm501176j.

[ref13] Xie D.; McCusker L. B.; Baerlocher C.; Zones S. I.; Wan W.; Zou X. SSZ-52, a Zeolite with an 18-Layer Aluminosilicate Framework Structure Related to That of the DeNO_x_ Catalyst Cu-SSZ-13. J. Am. Chem. Soc. 2013, 135 (28), 10519–10524. 10.1021/ja4043615. 23782259

[ref14] Kang J. H.; Walter R.; Xie D.; Davis T.; Chen C.-Y.; Davis M. E.; Zones S. I. Further Studies on How the Nature of Zeolite Cavities That Are Bounded by Small Pores Influences the Conversion of Methanol to Light Olefins. ChemPhysChem 2018, 19 (4), 412–419. 10.1002/cphc.201701197. 29211929

[ref15] Smeets S.; Zones S. I.; Xie D.; Palatinus L.; Pascual J.; Hwang S.-J.; Schmidt J. E.; McCusker L. B. SSZ-27: A Small-Pore Zeolite with Large Heart-Shaped Cavities Determined by Using Multi-Crystal Electron Diffraction. Angew. Chem., Int. Ed. 2019, 58 (37), 13080–13086. 10.1002/anie.201905049. PMC6773097 31347746

[ref16] Pérez-Ramírez J.; Christensen C. H.; Egeblad K.; Christensen C. H.; Groen J. C. Hierarchical Zeolites: Enhanced Utilisation of Microporous Crystals in Catalysis by Advances in Materials Design. Chem. Soc. Rev. 2008, 37 (11), 2530–2542. 10.1039/b809030k. 18949124

[ref17] Schwieger W.; Machoke A. G.; Weissenberger T.; Inayat A.; Selvam T.; Klumpp M.; Inayat A. Hierarchy Concepts: Classification and Preparation Strategies for Zeolite Containing Materials with Hierarchical Porosity. Chem. Soc. Rev. 2016, 45 (12), 3353–3376. 10.1039/C5CS00599J. 26477329

[ref18] Chen L.-H.; Sun M.-H.; Wang Z.; Yang W.; Xie Z.; Su B.-L. Hierarchically Structured Zeolites: From Design to Application. Chem. Rev. 2020, 120 (20), 11194–11294. 10.1021/acs.chemrev.0c00016. 32915551

[ref19] Davis M. E.; Saldarriaga C.; Montes C.; Garces J.; Crowdert C. A Molecular Sieve with Eighteen-Membered Rings. Nature 1988, 331 (6158), 698–699. 10.1038/331698a0.

[ref20] Paillaud J.-L.; Harbuzaru B.; Patarin J.; Bats N. Extra-Large-Pore Zeolites with Two-Dimensional Channels Formed by 14 and 12 Rings. Science 2004, 304 (5673), 990–992. 10.1126/science.1098242. 15143276

[ref21] Jiang J.; Xu Y.; Cheng P.; Sun Q.; Yu J.; Corma A.; Xu R. Investigation of Extra-Large Pore Zeolite Synthesis by a High-Throughput Approach. Chem. Mater. 2011, 23 (21), 4709–4715. 10.1021/cm201221z.

[ref22] Li J.; Corma A.; Yu J. Synthesis of New Zeolite Structures. Chem. Soc. Rev. 2015, 44 (20), 7112–7127. 10.1039/C5CS00023H. 25740693

[ref23] Roth W. J.; Nachtigall P.; Morris R. E.; Čejka J. Two-Dimensional Zeolites: Current Status and Perspectives. Chem. Rev. 2014, 114 (9), 4807–4837. 10.1021/cr400600f. 24555638

[ref24] Eliášová P.; Opanasenko M.; Wheatley P. S.; Shamzhy M.; Mazur M.; Nachtigall P.; Roth W. J.; Morris R. E.; Čejka J. The ADOR Mechanism for the Synthesis of New Zeolites. Chem. Soc. Rev. 2015, 44 (20), 7177–7206. 10.1039/C5CS00045A. 25946705

[ref25] Willhammar T.; Burton A. W.; Yun Y.; Sun J.; Afeworki M.; Strohmaier K. G.; Vroman H.; Zou X. EMM-23: A Stable High-Silica Multidimensional Zeolite with Extra-Large Trilobe-Shaped Channels. J. Am. Chem. Soc. 2014, 136 (39), 13570–13573. 10.1021/ja507615b. 25198917

[ref26] Corma A.; Díaz-Cabañas M. J.; Jordá J. L.; Martínez C.; Moliner M. High-Throughput Synthesis and Catalytic Properties of a Molecular Sieve with 18- and 10-Member Rings. Nature 2006, 443 (7113), 842–845. 10.1038/nature05238. 17051215

[ref27] Sun J.; Bonneau C.; Cantín Á.; Corma A.; Díaz-Cabañas M. J.; Moliner M.; Zhang D.; Li M.; Zou X. The ITQ-37 Mesoporous Chiral Zeolite. Nature 2009, 458 (7242), 1154–1157. 10.1038/nature07957. 19407798

[ref28] Corma A.; Díaz-Cabañas M. J.; Jiang J.; Afeworki M.; Dorset D. L.; Soled S. L.; Strohmaier K. G. Extra-Large Pore Zeolite (ITQ-40) with the Lowest Framework Density Containing Double Four- and Double Three-Rings. Proc. Natl. Acad. Sci. U.S.A. 2010, 107 (32), 13997–14002. 10.1073/pnas.1003009107. 20660773 PMC2922589

[ref29] Jiang J.; Jorda J. L.; Diaz-Cabanas M. J.; Yu J.; Corma A. The Synthesis of an Extra-Large-Pore Zeolite with Double Three-Ring Building Units and a Low Framework Density. Angew. Chem., Int. Ed. 2010, 49 (29), 4986–4988. 10.1002/anie.201001506. 20544765

[ref30] Martínez-Franco R.; Moliner M.; Yun Y.; Sun J.; Wan W.; Zou X.; Corma A. Synthesis of an Extra-Large Molecular Sieve Using Proton Sponges as Organic Structure-Directing Agents. Proc. Natl. Acad. Sci. U.S.A. 2013, 110 (10), 3749–3754. 10.1073/pnas.1220733110. 23431186 PMC3593851

[ref31] Jiang J.; Jorda J. L.; Yu J.; Baumes L. A.; Mugnaioli E.; Diaz-Cabanas M. J.; Kolb U.; Corma A. Synthesis and Structure Determination of the Hierarchical Meso-Microporous Zeolite ITQ-43. Science 2011, 333 (6046), 1131–1134. 10.1126/science.1208652. 21868673

[ref32] Möller K.; Bein T. Mesoporosity – a New Dimension for Zeolites. Chem. Soc. Rev. 2013, 42 (9), 3689–3707. 10.1039/c3cs35488a. 23460052

[ref33] Yuhas B. D.; Mowat J. P. S.; Miller M. A.; Sinkler W. AlPO-78: A 24-Layer ABC-6 Aluminophosphate Synthesized Using a Simple Structure-Directing Agent. Chem. Mater. 2018, 30 (3), 582–586. 10.1021/acs.chemmater.7b04891.

[ref34] Yang W.; Fu W.; Yuan Z.; Wang Z.; Teng J.; Tao W.; Zhao S. Silicon- and Germanium-based SCM-25 molecular sieve, preparation method therefor, and use thereof. Patent WO/2021/004492, January 14, 2021.

[ref35] Gemmi M.; Mugnaioli E.; Gorelik T. E.; Kolb U.; Palatinus L.; Boullay P.; Hovmöller S.; Abrahams J. P. 3D Electron Diffraction: The Nanocrystallography Revolution. ACS Cent. Sci. 2019, 5 (8), 1315–1329. 10.1021/acscentsci.9b00394. 31482114 PMC6716134

[ref36] Huang Z.; Willhammar T.; Zou X. Three-Dimensional Electron Diffraction for Porous Crystalline Materials: Structural Determination and Beyond. Chem. Sci. 2021, 12 (4), 1206–1219. 10.1039/D0SC05731B. PMC8179196 34163882

[ref37] Simancas J.; Simancas R.; Bereciartua P. J.; Jorda J. L.; Rey F.; Corma A.; Nicolopoulos S.; Pratim Das P.; Gemmi M.; Mugnaioli E. Ultrafast Electron Diffraction Tomography for Structure Determination of the New Zeolite ITQ-58. J. Am. Chem. Soc. 2016, 138 (32), 10116–10119. 10.1021/jacs.6b06394. 27478889 PMC5261824

[ref38] Liu X.; Luo Y.; Mao W.; Jiang J.; Xu H.; Han L.; Sun J.; Wu P. 3D Electron Diffraction Unravels the New Zeolite ECNU-23 from the “Pure” Powder Sample of ECNU-21. Angew. Chem., Int. Ed. 2020, 59 (3), 1166–1170. 10.1002/anie.201912488. 31674090

[ref39] Luo Y.; Wang B.; Smeets S.; Sun J.; Yang W.; Zou X. Exploring Polycrystalline Materials: High-Throughput Phase Elucidation Using Serial Rotation Electron Diffraction. ChemRxiv, October 27, 2021, ver. 1. 10.33774/chemrxiv-2021-34v44. PMC10070184 36717616

[ref40] Cichocka M. O.; Ångström J.; Wang B.; Zou X.; Smeets S. High-Throughput Continuous Rotation Electron Diffraction Data Acquisition via Software Automation. J. Appl. Crystallogr. 2018, 51 (6), 1652–1661. 10.1107/S1600576718015145. 30546290 PMC6276279

[ref41] Gao Z.; Balestra S. R. G.; Li J.; Camblor M. A. HPM-16, a New Stable Interrupted Zeolite with a Multidimensional Mixed Medium-Large Pore System Containing Supercages. Angew. Chem., Int. Ed. 2021, 60 (37), 20249–20252. 10.1002/anie.202106734. PMC8456927 34309150

[ref42] Corma A.; Díaz-Cabañas M. J.; Martínez-Triguero J.; Rey F.; Rius J. A Large-Cavity Zeolite with Wide Pore Windows and Potential as an Oil Refining Catalyst. Nature 2002, 418 (6897), 514–517. 10.1038/nature00924. 12152074

[ref43] Verheyen E.; Joos L.; Van Havenbergh K.; Breynaert E.; Kasian N.; Gobechiya E.; Houthoofd K.; Martineau C.; Hinterstein M.; Taulelle F.; Van Speybroeck V.; Waroquier M.; Bals S.; Van Tendeloo G.; Kirschhock C. E. A.; Martens J. A. Design of Zeolite by Inverse Sigma Transformation. Nat. Mater. 2012, 11 (12), 1059–1064. 10.1038/nmat3455. 23085567

[ref44] Pulido A.; Sastre G.; Corma A. Computational Study of ^19^F NMR Spectra of Double Four Ring-Containing Si/Ge-Zeolites. ChemPhysChem 2006, 7 (5), 1092–1099. 10.1002/cphc.200500634. 16612799

[ref45] Kabsch W. XDS. Acta Crystallogr. 2010, D66 (2), 125–132. 10.1107/S0907444909047337. PMC2815665 20124692

[ref46] Wan W.; Sun J.; Su J.; Hovmöller S.; Zou X. Three-Dimensional Rotation Electron Diffraction: Software RED for Automated Data Collection and Data Processing. J. Appl. Crystallogr. 2013, 46 (6), 1863–1873. 10.1107/S0021889813027714. 24282334 PMC3831301

[ref47] Sheldrick G. M. SHELXT – Integrated Space-Group and Crystal-Structure Determination. Acta Crystallogr. 2015, A71 (1), 3–8. 10.1107/S2053273314026370. PMC4283466 25537383

[ref48] Čejka J. Introduction to Zeolite Science and Practice; Elsevier Science & Technology Books, 2007.

[ref49] Hübschle C. B.; Sheldrick G. M.; Dittrich B. ShelXle: A Qt Graphical User Interface for SHELXL. J. Appl. Crystallogr. 2011, 44 (6), 1281–1284. 10.1107/S0021889811043202. 22477785 PMC3246833

[ref50] Smeets S.; McCusker L. B.; Baerlocher C.; Elomari S.; Xie D.; Zones S. I. Locating Organic Guests in Inorganic Host Materials from X-Ray Powder Diffraction Data. J. Am. Chem. Soc. 2016, 138 (22), 7099–7106. 10.1021/jacs.6b02953. 27181421

[ref51] Smeets S.. Topas Tools; Zenodo, 2021. 10.5281/zenodo.4719229.

[ref52] Coelho A. A. TOPAS and TOPAS-Academic: An Optimization Program Integrating Computer Algebra and Crystallographic Objects Written in C++. J. Appl. Crystallogr. 2018, 51 (1), 210–218. 10.1107/S1600576718000183.

[ref53] Blatov V. A.; Shevchenko A. P.; Proserpio D. M. Applied Topological Analysis of Crystal Structures with the Program Package ToposPro. Cryst. Growth Des. 2014, 14 (7), 3576–3586. 10.1021/cg500498k.

[ref54] BIOVIA Materials Studio; Dassault Systems. https://www.3ds.com/products-services/biovia/products/molecular-modeling-simulation/biovia-materials-studio/.

[ref55] Kasneryk V.; Shamzhy M.; Opanasenko M.; Wheatley P. S.; Morris S. A.; Russell S. E.; Mayoral A.; Trachta M.; Čejka J.; Morris R. E. Expansion of the ADOR Strategy for the Synthesis of Zeolites: The Synthesis of IPC-12 from Zeolite UOV. Angew. Chem., Int. Ed. 2017, 56 (15), 4324–4327. 10.1002/anie.201700590. PMC5396290 28295998

[ref56] Zhang J.; Veselý O.; Tošner Z.; Mazur M.; Opanasenko M.; Čejka J.; Shamzhy M. Toward Controlling Disassembly Step within the ADOR Process for the Synthesis of Zeolites. Chem. Mater. 2021, 33 (4), 1228–1237. 10.1021/acs.chemmater.0c03993.

[ref57] Mazur M.; Wheatley P. S.; Navarro M.; Roth W. J.; Položij M.; Mayoral A.; Eliášová P.; Nachtigall P.; Čejka J.; Morris R. E. Synthesis of ‘Unfeasible’ Zeolites. Nat. Chem. 2016, 8 (1), 58–62. 10.1038/nchem.2374. 26673264

