# Supplementary material for: SCM-25: A Zeolite with Ordered Meso-cavities Interconnected by 12 × 12 × 10-Ring Channels Determined by 3D Electron Diffraction
Source: Inorg Chem. 2022 Jan 25;61(10):4371–7. doi: 10.1021/acs.inorgchem.1c03632 (PMC8924920; doi:10.1021/acs.inorgchem.1c03632)
Supplement: Supplementary file 1 — ic1c03632_si_001.pdf [file ic1c03632_si_001.pdf]

Supplementary materials for

SCM-25: a zeolite with ordered meso-cavities interconnected by 12×12×10-ring channels determined by 3D electron diffraction

Yi Luo[1†], Wenhua Fu[2†], Bin Wang[1], Zhiqing Yuan[2], Junliang Sun[1,3]*, Xiaodong Zou[1]* and Weimin Yang[2]*,

[1]Department of Materials and Environmental Chemistry, Stockholm University, SE-106 91 Stockholm, Sweden.

[2]State Key Laboratory of Green Chemical Engineering and Industrial Catalysis, Sinopec Shanghai Research Institute of Petrochemical Technology, 1658 Pudong Beilu, Shanghai 201208, China.

[3]College of Chemistry and Molecular Engineering, Beijing National Laboratory for Molecular Sciences, Peking University, Beijing 100871, China.

[†]Y. Luo and W. Fu contributed equally to this work.

[*]Corresponding authors: W. Y. (yangwm.sshy@sinopec.com)

X. Z. (xzou@mmk.su.se)

J. S. (junliang.sun@pku.edu.cn)

Table of Contents

Materials and Methods…..……...3
Figure S1...6
Figure S2...7
Figure S3...8
Figure S4...8
Figure S5...9
Figure S6...10
Figure S7...11
Figure S8...12
Figure S9...13
Figure S10...14
Figure S11...15
Figure S12...16
Figure S13...17
Figure S14...18
Figure S15...19
Figure S16...20
Figure S17...21
Table S1…………..22
Table S2…………..23
Table S3…………..24
Table S4…………..25
Table S5…………..26
Table S6…………..27
References...28

Materials and Methods

Materials

Germanosilicate zeolite SCM-25 was synthesized using 1,1,3,5-tetramethyl piperidinium hydroxide (1,1,3,5-TMPOH, 20 wt%, SACHEM Inc.) as organic structure-directing agent (OSDA) in fluoride medium. In a typical synthesis of SCM-25, 0.7 g germanium oxide (GeO_2, 99.0%, Sinopharm Chemical Reagent Co., Ltd) and 2.78 g tetraethyl orthosilicate (TEOS, AR, Sinopharm Chemical Reagent Co., Ltd) were added to 7.78 g aqueous solution of 1,1,3,5-TMPOH. The mixture was stirred at room temperature until the evaporation of all ethanol formed during the hydrolysis of TEOS and some water. Then, 0.5 g hydrofluoric acid (HF, 40 wt%, Sinopharm Chemical Reagent Co., Ltd, **Caution! highly toxic**) was added. In the end, some water was introduced in order to obtain a synthesis gel with a molar composition of 0.5 1,1,3,5-TMPOH: 0.667 SiO_2: 0.333 GeO_2: 0.5 HF: 7 H_2O. The resulting gel was transferred into a Teflon-lines steel autoclave and heating in a rotating oven (20 rpm) at 175 ºC for 14 days. The solids were recovered by centrifugation, washing, and drying (373 K, 12h).

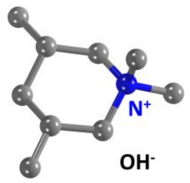

Molecular structure of 1,1,3,5-TMPOH

Methods

1. 3D ED data collection and structure refinement of SCM-25 against the 3D ED data.

The 3D ED data was collected using the continuous rotation (cRED) method implemented in software *Instamatic*, which is installed in a JEOL JEM2100 TEM (LaB6 filament, ASI Timepix camera) operating at 200 kV.[1]

The kinematical structure refinement of SCM-25 against 3D ED data was conducted using the program *SHELXLE*.[2] The atomic scattering factors (eight parameters) of electrons were sued. The disordered atoms in the framework structure were refined with bond and angle restraints using the commands AFIX, DANG, and RIGU in SHELXLE. All the other atoms were freely refined. The atoms were firstly refined isotropically. After the refinement converged, the atoms were then further refined anisotropically with the gradually released DAMP parameter.

2. Synchrotron powder X-ray diffraction (PXRD) characterization and Rietveld refinement.

Synchrotron PXRD data were collected on the as-made SCM-25 in a 0.8 mm capillary on the beamline 11-BM-B (λ= 0.412836 Å) at the Advanced Photon Source, Illinois, USA. The collected data were ranging from 0.700 to 20.000° with 0.001° data binning.

The Rietveld refinement of SCM-25 against synchrotron PXRD data was conducted using the program Topas 6.0 and topas_tools.[3,4] The OSDAs were included in refinement. The OSDA molecule was generated and optimized using the molecular modelling algorithm implemented in Materials Studio 7.0.[5] As indicated by the electron density difference map generated against the synchrotron PXRD data, four symmetry-independent positions were revealed for OSDA molecules. Therefore, four OSDA molecules constrained as rigid bodies were introduced in the structure model of SCM-25. The hydrogen atoms of the OSDA molecules were omitted in the refinement, but their contributions were counted on their connecting carbon atoms. The occupancies and positions of the four OSDA molecules were then refined using the simulated-annealing algorithm implemented in TOPAS 6.0.[3] The total number of OSDA molecules per unit cell was constrained at 10.0, as indicated by TGA results (Figure S4 and Table S1).

3. Other characterizations

In house powder X-ray diffraction (PXRD) patterns were collected on a PANalytical X'Pert PRO diffractometer with Cu *Kα* radiation, operated at 40 kV and 40 mA in the 2θ range of 5°-40°. In-situ PXRD patterns were obtained on a Bruker D8 Advance X-ray diffractometer with Cu Kα as the radiation source at an operating voltage of 40 kV and current of 40 mA, through direct in situ calcination of the as-made SCM-25 zeolite sample in an Anton Parr XRK-900 chamber, at a heating rate of 10 °C min^{-1}. Scanning electron microscopy (SEM) measurements were performed on a Hitachi S-4800 field-emission scanning electron microscope (Hitachi, Japan) with an acceleration voltage of 3 kV. Thermogravimetric analysis (TGA) was measured on a TA SDT-Q600 instrument by heating the samples to 800 °C at a rate of 10 °C/min. Nitrogen adsorption isotherms at 77 K were measured on a Quantachrome Quadrasorb Evo volumetric adsorption analyzer. The samples were outgassed at 573 K for 6 h before the measurements. The Brunauer-Emmet-Teller (BET) specific surface area was calculated based on the adsorption data acquired in a relative pressure (p/p_0) range of 0.01-0.1, and the total pore volume was determined from the amounts adsorbed at a relative pressure of about 0.995. Argon adsorption isotherm at 77 K was recorded on a Micromeritics ASAP 2020 adsorption instrument. Inductively coupled plasma-atomic emission spectrometry (ICP-AES) measurements were performed using a Thermo IRIS Intrepid II XSP atomic emission spectrometer, after dissolving the samples in HF solution. ^{29}Si, ^{19}F and ^{13}C magic angle spinning nuclear magnetic resonance (MAS NMR)

measurements were performed using a Varian-400 spectrometer. ^{29}Si NMR spectra were obtained at 79.4 MHz using a 4-mm MAS probe with a spinning rate of 10 kHz. For ^{1}H-^{29}Si CP/MAS experiments, the contact time was fixed to 5 ms, and the delay between consecutive pulses was 4s. ^{19}F NMR spectrum was obtained at 376.3 MHz using a 2.5-mm MAS probe with a spinning rate of 30 kHz. ^{13}C NMR spectrum was recorded with a 7.5 mm probe at 100.54 MHz and a spinning rate of 5 kHz.

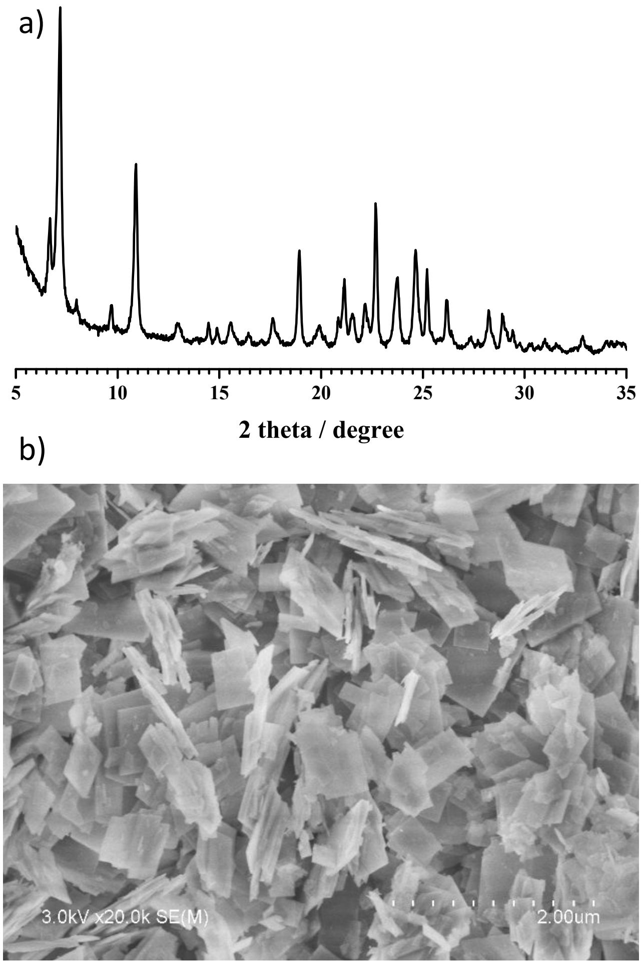

Figure S1 PXRD pattern (a) and SEM image (b) of as-made SCM-25.

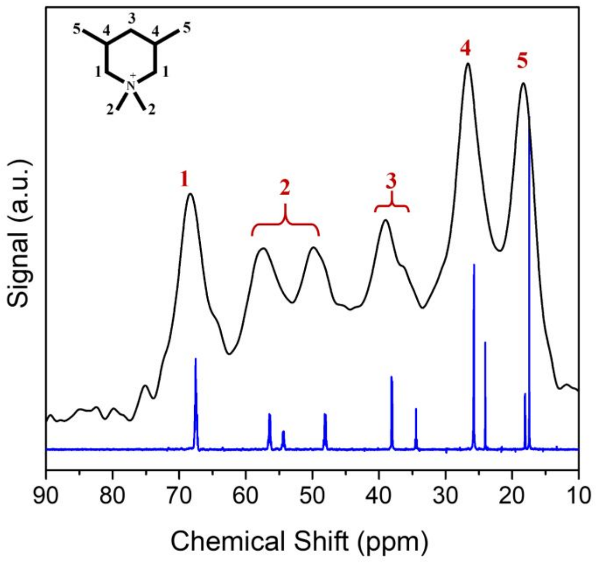

Figure S2 Comparison of liquid state ^{13}C NMR spectrum of TMPOH aqueous solution (bottom, blue curve) and solid-state ^{13}C MAS NMR spectrum of as-made SCM-25 (top, black curve), which shows that the OSDAs remain intact in the structure of SCM-25.[6]

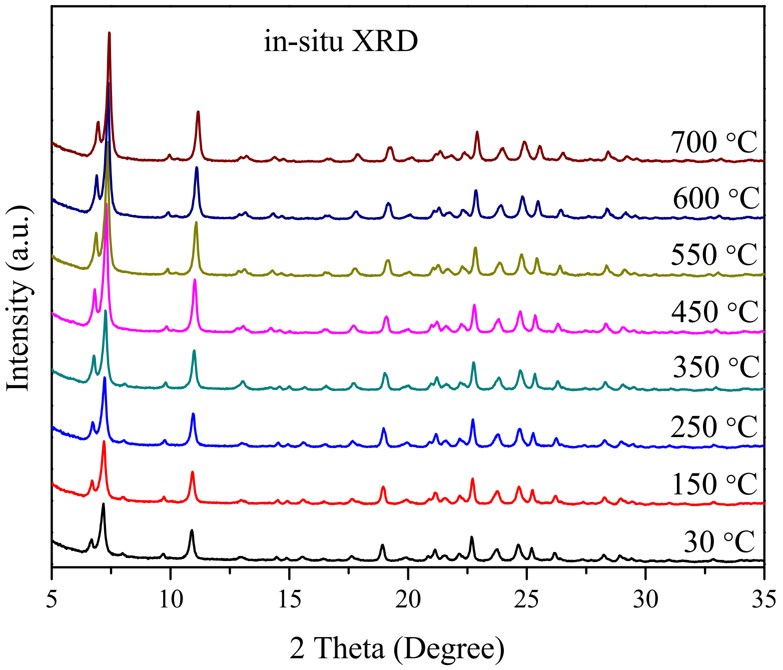

Figure S3 In-situ PXRD patterns of as-made SCM-25 heating at different temperatures. The framework structure of SCM-25 is stable after being heated at 700°C. The increasing intensities of the reflections at the low angle region are mainly attributed to removing OSDAs in channels.

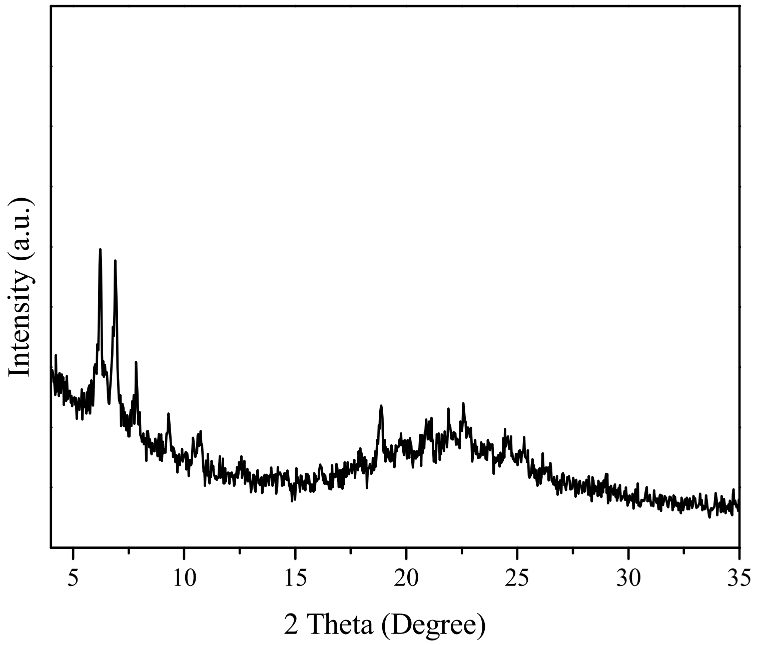

Figure S4 PXRD pattern of calcined SCM-25 (900°C). The framework structure of SCM-25 has partially collapsed after being calcined at 900°C.

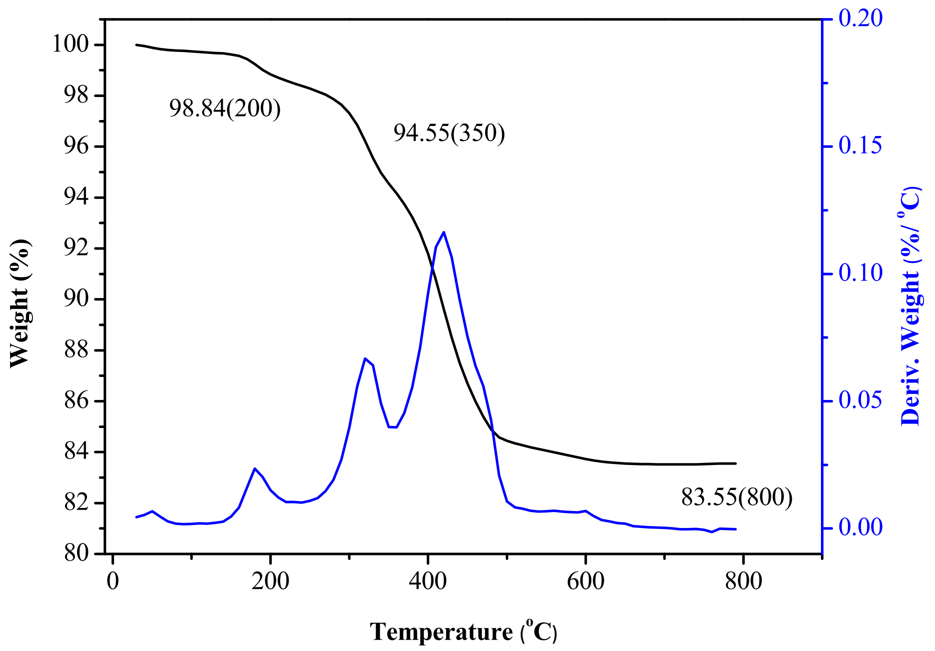

Figure S5 TGA-DSC curves of as-made SCM-25. The weight lost before 200℃ was assigned to water. While the weight loss between 200-800℃ could be attributed to OSDAs and F^-. The chemical composition of SCM-25 calculated based on the TGA curve was summarized in Table S1.

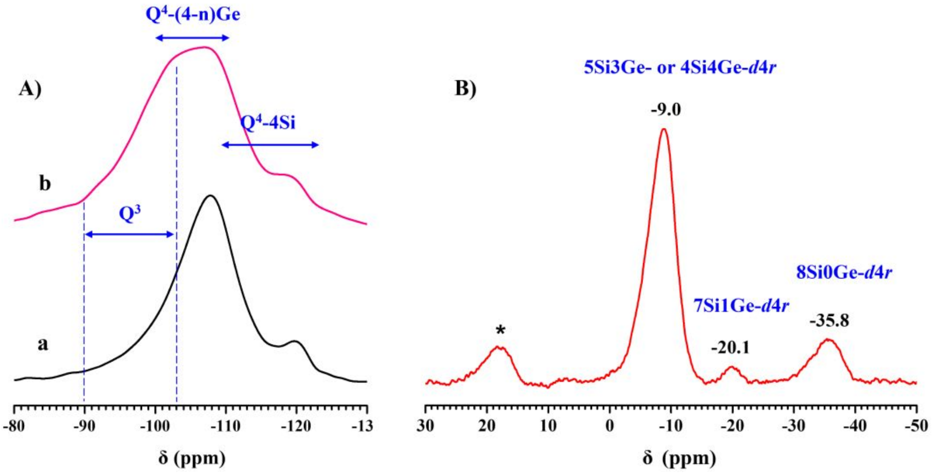

Figure S6 A) ^{29}Si MAS NMR spectrum (a) and ^{1}H to ^{29}Si CPMAS-NMR spectrum of calcined SCM-25 (b). B) ^{19}F MAS NMR spectrum of as-made SCM-25. Spinning sidebands are marked with asterisks.

The ^{29}Si MAS NMR spectrum of the calcined SCM-25 shows that most Si atoms are four coordinated (Q^4 species, $Si(OSi)_4$). Q^4 silicon atoms with at least one directly connected neighbouring Ge atom (Q^4-nGe) show a chemical shift in the range of -100 to -110 ppm. Chemical shifts at -110 to 120 ppm are assigned to the Q^4 silicon atoms that are only surrounded by silicon atoms. The chemical shifts at -90 to -102 ppm are normally attributed to the Q^3 species of silicon atoms ($Si(OSi)_3OH$), which indicates the defects (silanols) in the framework structure.[7-9] The enhanced signal corresponding in the ^{1}H to ^{29}Si CPMAS-NMR spectrum has also confirmed the silanol defects. These results are all consistent with the framework structure resolved by 3D ED.

Three typical resonance bands centered at -9.0, -20.1, and -35.8 ppm presented in the ^{19}F solid-state MAS NMR spectrum confirmed the existence of double 4-ring (*d*4*r*) units in the framework structure.[10] The chemical shifts of those three bands indicate the approximate T atoms composition of *d*4*r* units could be $Si_{4.0}Ge_{4.0}$ or $Si_{5.0}Ge_{3.0}$, $Si_{7.0}Ge_{1.0}$, and $Si_{8.0}Ge_{0.0}$ correspondingly.[10] The dominated band at -9.0 ppm demonstrating the major compositions of *d*4*r* units are closed to $Si_{4.0}Ge_{4.0}$ or $Si_{5.0}Ge_{3.0}$, which is in good agreement with the averaged chemical composition of *d*4*r* units that refined against the 3D ED data.

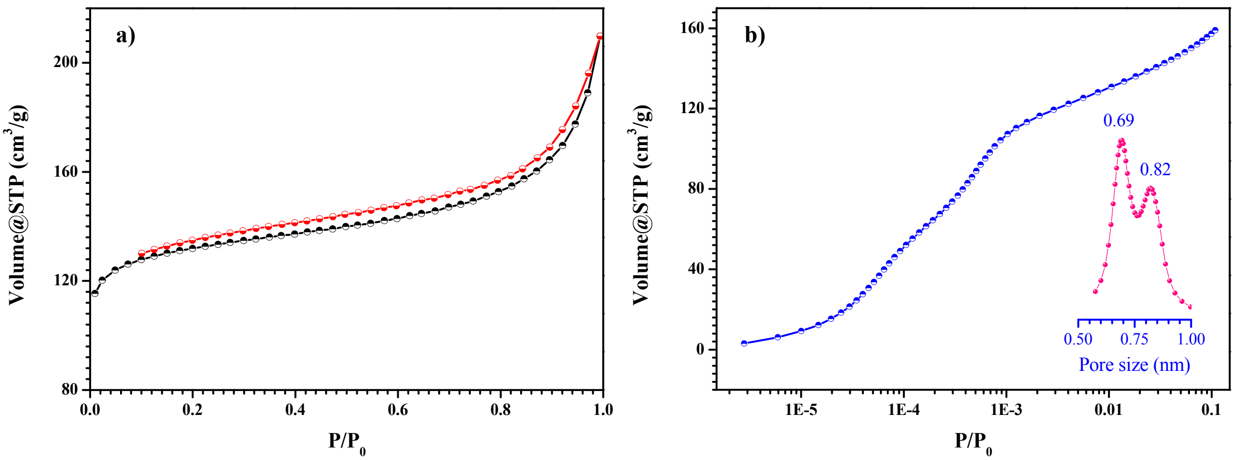

Figure S7 a) N_2 adsorption/desorption isotherm, and b) Ar adsorption isotherm and corresponding pore size distribution.

The N_2 adsorption measurements give a BET surface area of 511 m^2/g with a micropore volume of 0.18 cm^3/g. Meanwhile, the Ar adsorption measurements show a bimodal pore size distribution centered at 6.9 and 8.2 Å (calculated by the Horváth-Kawazoe method), which reveals the medium and large pores in SCM-25.[11]

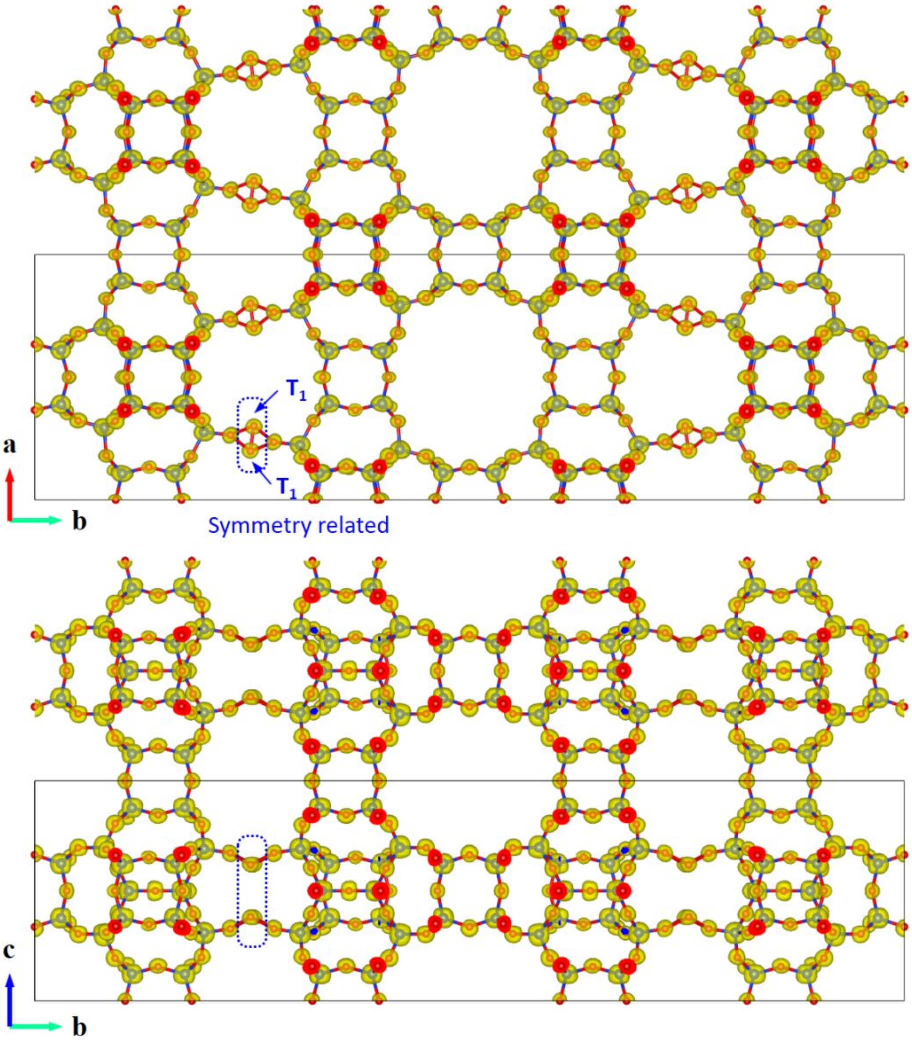

Figure S8 Observed electron density map of the initial structure model (2×1×2 unit cells) of as-made SCM-25 resolved from 3D ED data. Most of the atoms in the asymmetric unit including a disordered T atom (donated as T_1 in blue frame) were identified directly. The location of T_1 is a position for Si or Ge atom but has been assigned to an O atom by *SHELXT*. This is because T_1 atoms located there are half occupied. Based on the basic principle of zeolite chemistry, the defects and missing O atoms related to the disordered T_1 atoms could be resolved straightforwardly.

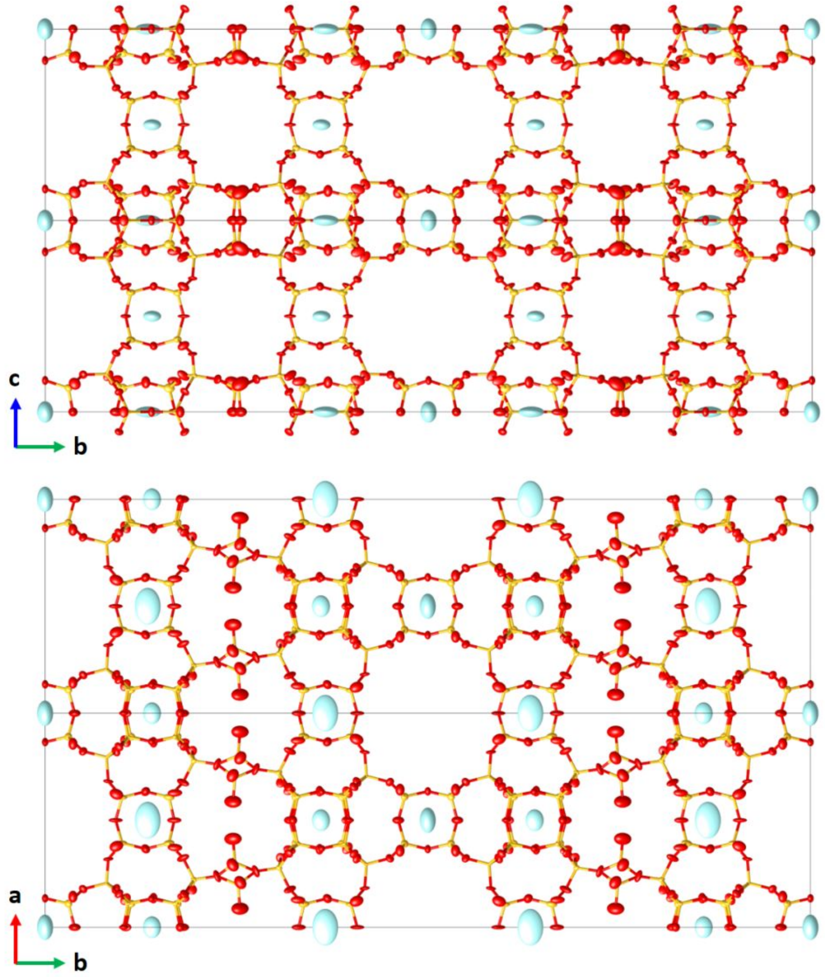

Figure S9 Crystal structural model of as-made SCM-25 shown in thermal ellipsoids refined against 3D ED data (OSDAs were not included in the refinement). The atomic displacement parameters of all framework atoms are reasonable, which shows accurate structure of SCM-25 could be obtained from the 3D ED data.

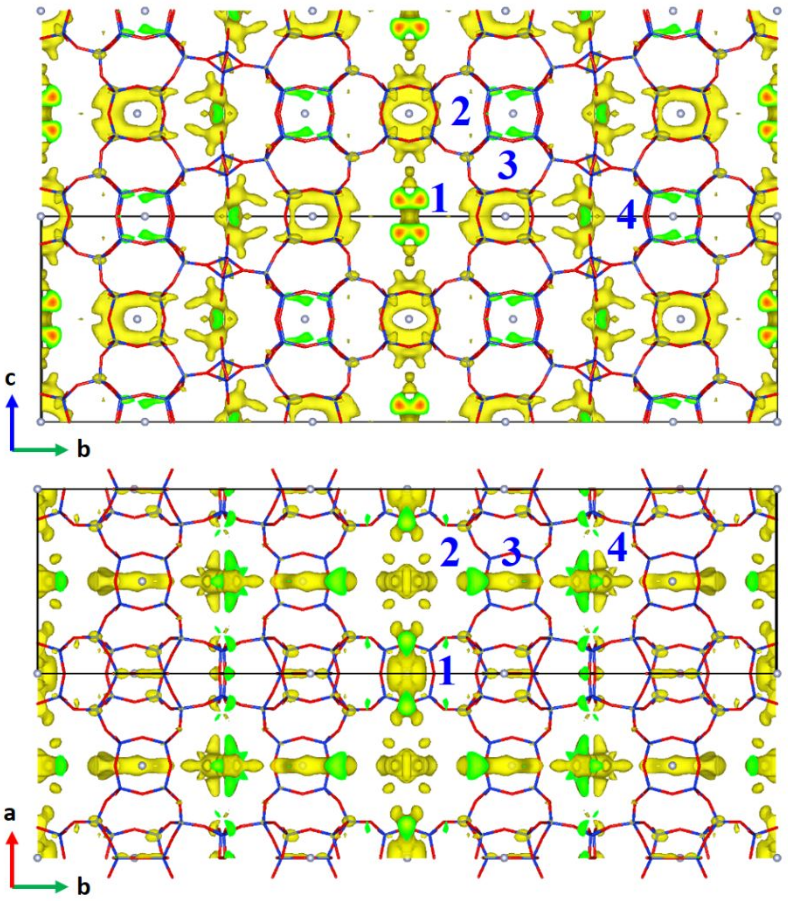

Figure S10 The difference electron density map of as-made SCM-25 generated based on the synchrotron PXRD data. Four symmetry-independent locations of OSDAs could be clearly identified (marked 1-4) based on the residual electron density map in the channels of SCM-25.

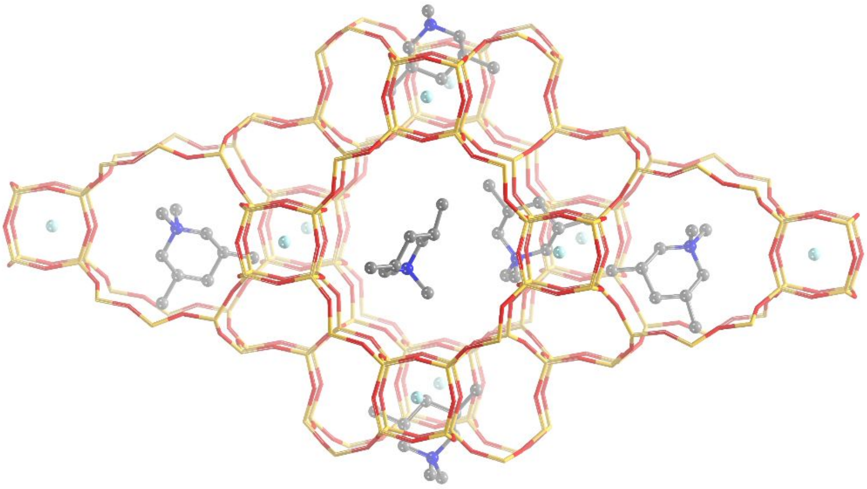

Figure S11 A type of possible ordered distribution of OSDAs in the meso-cavities. They show a trend to approach the *d*4*r* units (where the F- ions are located) and the part of the framework where Van der Waals interactions may occur. Electrostatic and Van der Waals interactions may therefore play essential roles in the formation of SCM-25.

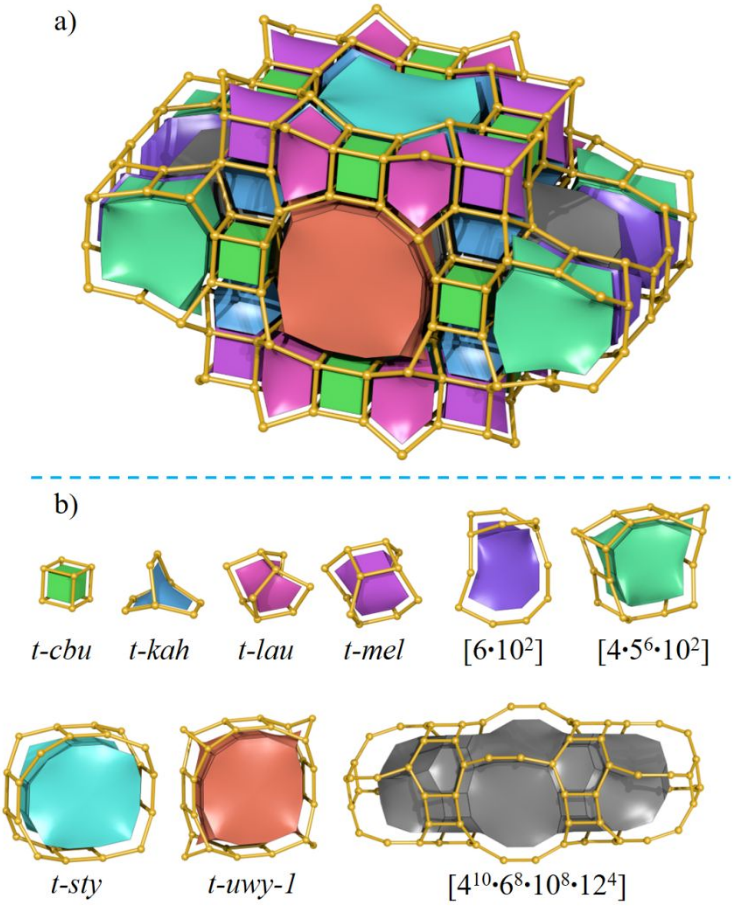

Figure S12 Topology analysis of SCM-25. a) The natural tilling composition of SCM-25, b) different tiles in the framework of SCM-25. The large complex tiles with a face symbol of [4^{10}•6^8•10^8•12^4] disclosure unique shuttle-like meso-cavities in SCM-25.

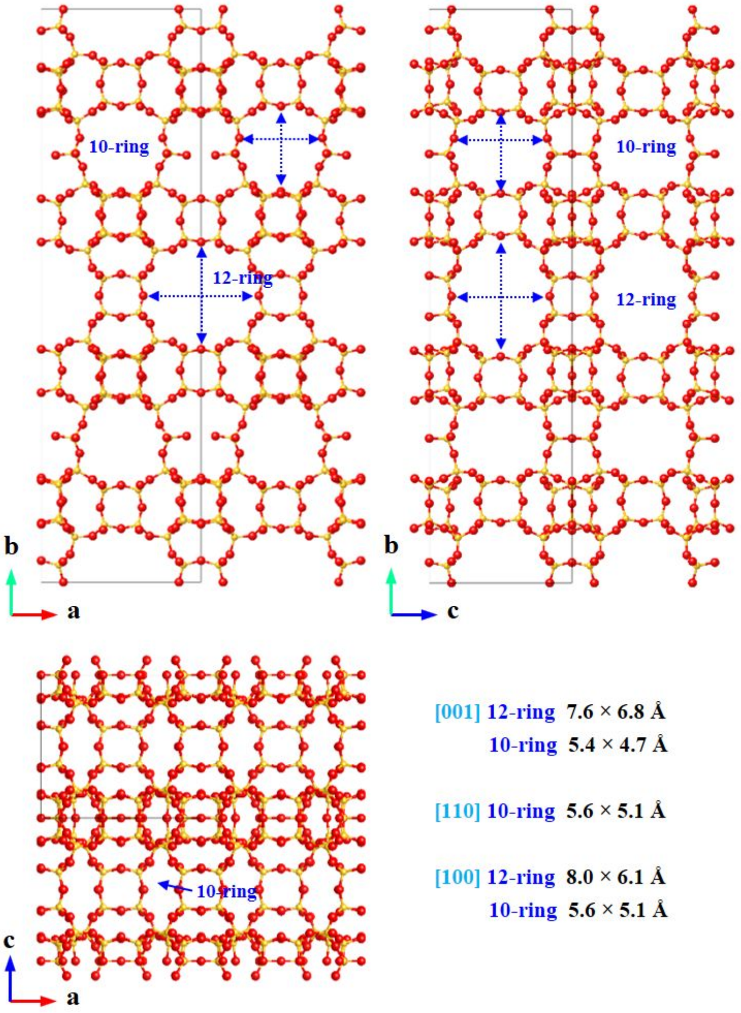

Figure S13 One type of ordered framework structure SCM-25 (2×2×2 unit cells) and its pore openings along different directions. The sizes of pore opening are calculated by taking into account an oxygen radius of 1.35 Å.

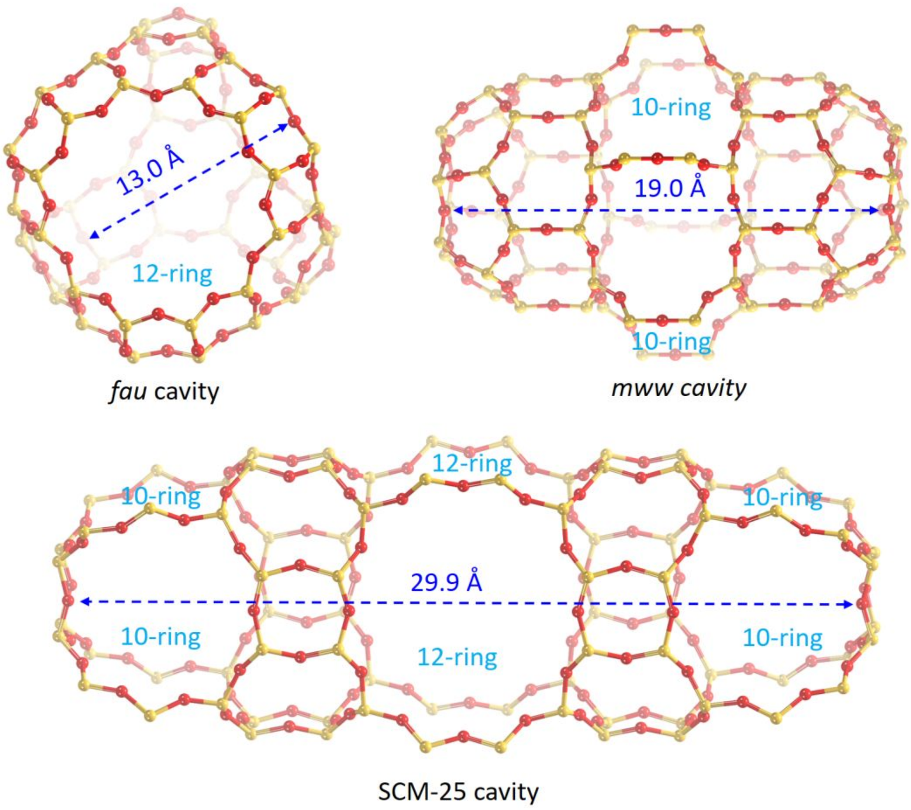

Figure S14 The cavities in FAU (3D, 12×12×12-ring), MWW (2D, 10×10-ring), and SCM-25 (3D, 12×12×10-ring). The sizes of pore opening are calculated by taking into account an oxygen radius of 1.35 Å.

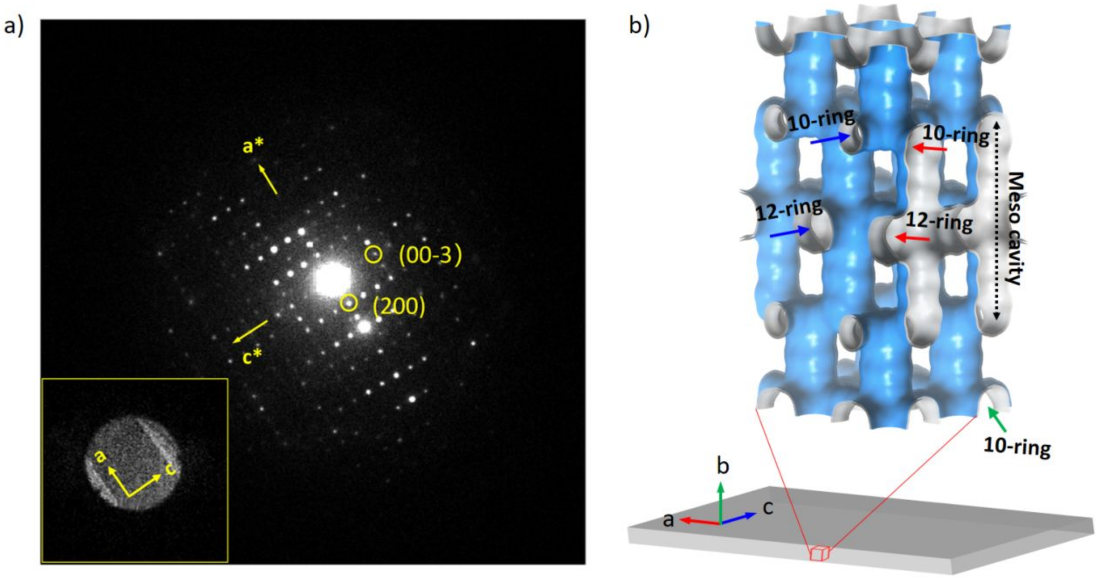

Figure S15 a) A single diffraction pattern acquired over a plate-like crystal of SCM-25. The indexing results indicate that the *b* axis is perpendicular to the plate-like crystal, the *a* axis is along the longest direction of the crystal, and the *c* axis is the second-longest direction of the crystal. b) The distribution of the channel system in the crystals of SCM-25. The longest direction of the meso-cavities is perpendicular to the plate-like crystals.

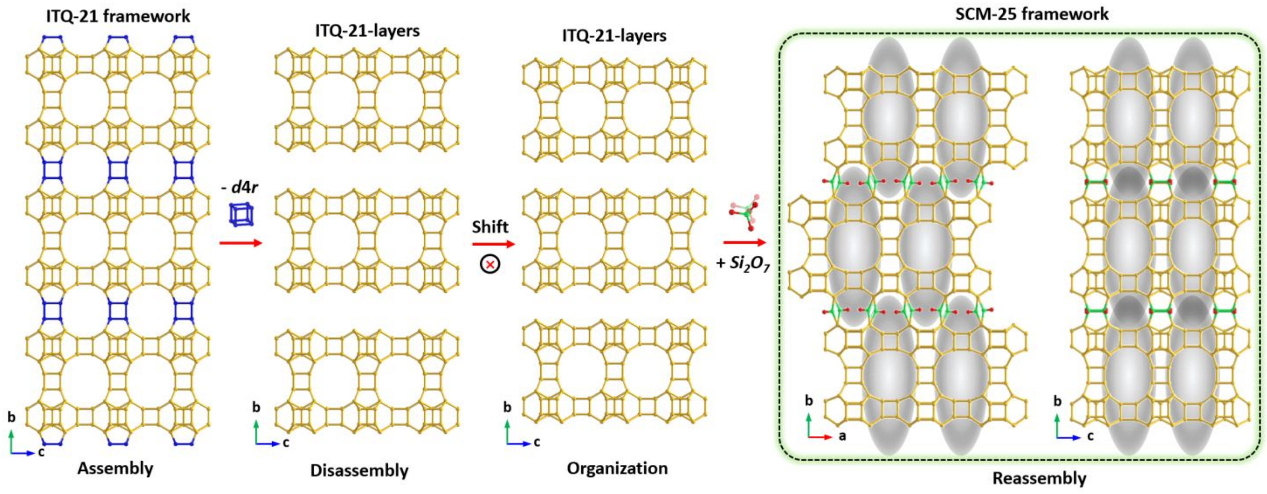

Figure S16 Structural relationship between ITQ-21 and SCM-25 viewed along the *a*-axis. The framework structure of ITQ-21 can be transformed into the framework structure of SCM-25 via the ADOR approach (the shift is along the a-axis). It is worth mentioning while the single 4-ring (*s*4*r*) in ITQ-21 is disordered with three possible orientations perpendicular to *a*, *b* or *c*-axis, respectively (here the *s*4*r* is shown to be perpendicular to the *c*-axis), it is ordered in SCM-25.

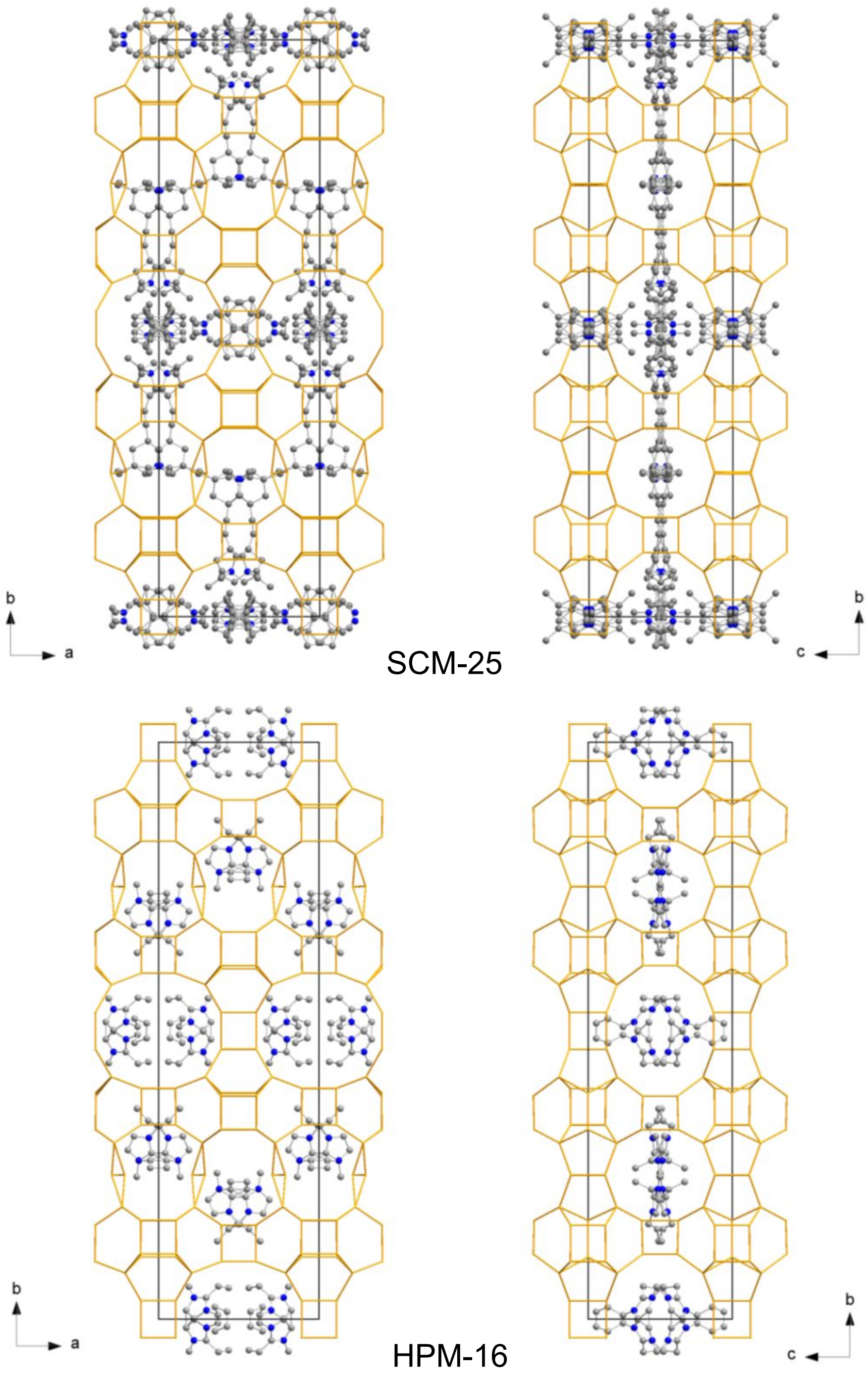

Figure S17 Comparison of the locations of the OSDAs in SCM-25 (top) and HPM-16 (bottom). Four symmetry independent 1,1,3,5-TMP^{+} cations are located in SCM-25, and two symmetry independent 1M2E3nPrIM^{+} cations are found in HPM-16. All OSDAs have partial occupancies and are disordered in several positions (all are shown). Carbon atoms are shown in grey and nitrogen in blue. Hydrogen atoms are omitted for clarity.

Table S1 Chemical Composition of as-made SCM-25

sample	ICP	TGA analysis		Calculated chemical composition
	Si/Ge	H_2O/u.c.	(OSDA+F)/u.c.	
SCM-25	2.4	7.76	10.58	$\|(C_9NH_{20}F)_{10.6}\ (H_2O)_{7.8}\|[Si_{96}Ge_{40}O_{276}]$

Table S2 Experimental parameters of cRED and Crystallographic data of the structure of as-made SCM-25

**Data collection**	
Sample	**SCM-25**
Tilt range (°)	-65.59 ~ 25.02
Tilt step (°)	0.23
Wave length (Å)	0.0251
No. of frames	342
Exposure time per image (s)	0.5
Total time of data collection (min)	3.4
**Unit cell determined by XDS**	
Crystal system	orthorhombic
Space group	*C*222
a, *b*, *c* (Å)	14.62, 51.82, 13.11,
α, *β*, *γ* (°)	90, 90, 90
**Data details**	
Resolution (Å)	0.80
$I/\sigma(I)$	3.70
Completeness (%)	89.8
$CC_{1/2}$	99.6
R_{meas} (%)	15.6
No. of total reflections	14527
No. of unique reflections	5079
**Structure solution by *SHELXT***	
Crystal system	orthorhombic
space group	*Cmmm*
a, *b*, *c* (Å)	14.62, 51.82, 13.11,
α, *β*, *γ* (°)	90, 90, 90
No. of atoms in asymmetric unit	34
Initial wR_2 (%)	64.34
Initial R_1 (%)	30.48

Table S3 Crystallographic details of the structure refinement against the 3D ED data collected on as-made SCM-25.

**Crystal data**	
Formula	$\|F^{-}_{10}\|[Si_{94.7}Ge_{41.3}O_{276}]$
Formula Weight	10218.7
Crystal System	Orthorhombic
Space group	$Cmmm$ (No. 65)
a, b, c (Å)*	14.2510, 50.995, 12.735
α, β, γ (°)	90, 90, 90
V (Å^3)	9254.6
ρ (g/cm^3)	1.834
F(000)	1419
**Data details**	
Temperature (K)	293
Radiation (Å)	electrons, 0.02508
d_{max}, d_{min} (Å)	13.73, 0.80
Dataset (h, k, l)	-18→17, -63→63, -15→16
Tot., Uniq. data, R_{int}	14527, 5079, 0.156
Observed Data [$I > 4.0\sigma(I)$]	2283
**Refinement**	
$N_{reflections}$, $N_{parameters}$, $N_{restraints}$	5079, 286, 26
$R1$, $wR2$, S [$I > 4.0\sigma(I)$]	0.1848, 0.4388, 1.684
$(\Delta/\sigma)_{max}$	0.00
ρ_{min}, ρ_{max} (e$^-$/Å^3)	-0.419, 0.078

$N_{restraints}$=2 DFIX+6 DANG+18RIGU

*The unit cell parameters identified from PXRD data was applied in the refinement.

Table S4 Selected bond distances and angels for as-made SCM-25 refined against the 3D ED and synchrotron PXRD

data.

Selected bond distances (Å) and angles (°)	Theoretical values	Refined values					
		3D ED			PXRD		
		min.	max.	avg.	min.	max.	avg.
Si-O	1.61	1.57	1.62	1.60	1.58	1.64	1.61
(Si,Ge)-O	(1.61,1.74)	1.60	1.69	1.65	1.62	1.69	1.65
O-(Si)-O	(109.5)	104.3	114.6	109.5	105.8	112.0	109.5
O-(Si,Ge)-O	(109.5)	103.3	114.9	109.3	103.6	114.5	109.4
Si-O-Si	(145)	148.4	170.4	158.6	147.1	171.5	160.3
Si-O-(Si,Ge)	(145)	136.2	149.1	141.9	132.8	148.4	140.8
(Si,Ge)-O-(Si,Ge)	(145)	132.6	149.3	141.2	132.0	148.6	142.5

Table S5 Crystallographic details of the structure refinement against the PXRD data collected on as-made SCM-25.

Material	SCM-25
Composition	$|(C_9H_{20}N^+)(F^-)(H_2O)_{0.93}|_{10}[Si_{96}Ge_{40}O_{272}]$
Space group	*Cmmm*
a (Å)	14.25093(12)
b (Å)	50.9950(9)
c (Å)	12.73475(11)
V (Å^3)	9254.67(9)
2θ range (°)	0.7 to 20
Wavelength (Å)	0.412836
R_B	0.033
R_{wp}	0.170
R_{exp}	0.099
Observations	19331
Contributing reflections	1161
Parameters	194
Restraints	123

Table S6 Comparison of atomic positions and occupancies of SCM-25 refined against the 3D ED and PXRD data.

	3D ED				PXRD				Deviation
Atom	x	y	z	Occ.	x	y	z	Occ.	Δ(Å)[b]
Si01	0.1099	0.03021	0.1242	0.44[a]	0.1112	0.02985	0.1249	0.481[a]	0.0276
Si02	0.2056	0.08013	0.1972	1.0	0.2098	0.07870	0.2003	0.936[a]	0.1023
Si03	0.1093	0.11181	0.3769	0.42[a]	0.1122	0.11187	0.3762	0.501[a]	0.0424
Si04	0.1108	0.10825	0.0000	1.0	0.1107	0.10750	0.0000	0.932[a]	0.0383
Si05	0.1084	0.17250	0.3784	0.43[a]	0.1112	0.17286	0.3768	0.476[a]	0.0484
Si06	0.1106	0.17014	0.0000	1.0	0.1104	0.16980	0.0000	0.929[a]	0.0176
Si07	0.2231	0.19444	0.1895	1.0	0.2239	0.19470	0.1917	0.975[a]	0.0330
Si08	0.1068	0.39950	0.1235	0.54[a]	0.1052	0.40034	0.1243	0.597[a]	0.0496
Si09	0.1077	0.33819	0.1241	0.58[a]	0.1078	0.33869	0.1252	0.603[a]	0.0291
Si10	0.1978	0.25200	0.1230	0.5	0.2009	0.25170	0.1251	0.5	0.0539
O001	0.1349	0.00000	0.1642	1.0	0.1380	0.00000	0.1560	1.0	0.1134
O002	0.1428	0.03630	0.0000	1.0	0.1360	0.03570	0.0000	1.0	0.1016
O003	0.0000	0.03860	0.1472	1.0	0.0000	0.03750	0.1560	1.0	0.1253
O004	0.1781	0.04960	0.1946	1.0	0.1810	0.04850	0.1970	1.0	0.0761
O005	0.1788	0.09260	0.3073	1.0	0.1817	0.09160	0.3099	1.0	0.0735
O006	0.1816	0.41750	0.1891	1.0	0.1775	0.41940	0.1935	1.0	0.1263
O007	0.1566	0.09640	0.1040	1.0	0.1597	0.09510	0.1056	1.0	0.0822
O008	0.0000	0.10530	0.3449	1.0	0.0000	0.10330	0.3490	1.0	0.1146
O009	0.1310	0.10500	0.5000	1.0	0.1360	0.10530	0.5000	1.0	0.0729
O010	0.1357	0.14150	0.3446	1.0	0.1344	0.14230	0.3429	1.0	0.0498
O011	0.0000	0.10260	0.0000	1.0	0.0000	0.10100	0.0000	1.0	0.0816
O012	0.1329	0.13880	0.0000	1.0	0.1230	0.13870	0.0000	1.0	0.1412
O013	0.0000	0.40780	0.1642	1.0	0.0000	0.40650	0.1710	1.0	0.1091
O014	0.1216	0.40690	0.0000	1.0	0.1130	0.40880	0.0000	1.0	0.1562
O015	0.1339	0.36880	0.1543	1.0	0.1310	0.36960	0.1463	1.0	0.1173
O016	0.1387	0.18070	0.5000	1.0	0.1426	0.17940	0.5000	1.0	0.0865
O017	0.0000	0.18040	0.3455	1.0	0.0000	0.18210	0.3560	1.0	0.1594
O018	0.1763	0.19220	0.3030	1.0	0.1744	0.19220	0.3026	1.0	0.0276
O019	0.0000	0.17620	0.0000	1.0	0.0000	0.17750	0.0000	1.0	0.0663
O020	0.1560	0.18200	0.1053	1.0	0.1607	0.18190	0.1013	1.0	0.0843
O021	0.1277	0.33040	0.0000	1.0	0.1190	0.33040	0.0000	1.0	0.1240
O022	0.1766	0.31980	0.1902	1.0	0.1794	0.32090	0.1962	1.0	0.1028
O023	0.0000	0.33130	0.1594	1.0	0.0000	0.33080	0.1660	1.0	0.0878
O024	0.2386	0.22480	0.1705	1.0	0.2402	0.22490	0.1733	1.0	0.0426
O025	0.2090	0.25500	0.0000	0.5	0.2150	0.25200	0.0000	0.5	0.1753
O026	0.0869	0.25460	0.1470	0.5	0.0900	0.25500	0.1510	0.5	0.0704

[a] The rest is the occupancy of Ge atom (ie, 1-0.4). [b] $\Delta = \sqrt[2]{(\Delta x)^2 * a^2 + (\Delta y)^2 * b^2 + (\Delta z)^2 * c^2}$

References

(1) Cichocka, M. O.; Ångström, J.; Wang, B.; Zou, X.; Smeets, S. High-Throughput Continuous Rotation Electron Diffraction Data Acquisition via Software Automation. *J. Appl. Cryst.* **2018**, *51* (6), 1652-1661.

(2) Hübschle, C. B.; Sheldrick, G. M.; Dittrich, B. ShelXle: A Qt Graphical User Interface for SHELXL. *J. Appl. Cryst.* **2011**, *44* (6), 1281-1284.

(3) Coelho, A. A. TOPAS and TOPAS-Academic: An Optimization Program Integrating Computer Algebra and Crystallographic Objects Written in C++. *J. Appl. Cryst.* **2018**, *51* (1), 210-218.

(4) Stef Smeets. *Topas Tools*; Zenodo, 2021. https://doi.org/10.5281/zenodo.4719229.

(5) BIOVIA Materials Studio - BIOVIA - Dassault systems. https://www.3ds.com/products-services/biovia/products/molecular-modeling-simulation/biovia-materials-studio/.

(6) Dusselier, M.; Schmidt, J. E.; Moulton, R.; Haymore, B.; Hellums, M.; Davis, M. E. Influence of Organic Structure Directing Agent Isomer Distribution on the Synthesis of SSZ-39. *Chem. Mater.* **2015**, *27* (7), 2695-2702.

(7) Verheyen, E.; Joos, L.; Van Havenbergh, K.; Breynaert, E.; Kasian, N.; Gobechiya, E.; Houthoofd, K.; Martineau, C.; Hinterstein, M.; Taulelle, F.; Van Speybroeck, V.; Waroquier, M.; Bals, S.; Van Tendeloo, G.; Kirschhock, C. E. A.; Martens, J. A. Design of Zeolite by Inverse Sigma Transformation. *Nat. Mater.* **2012**, *11* (12), 1059-1064.

(8) Smeets, S.; Xie, D.; Baerlocher, C.; McCusker, L. B.; Wan, W.; Zou, X.; Zones, S. I. High-Silica Zeolite SSZ-61 with Dumbbell-Shaped Extra-Large-Pore Channels. *Angew. Chem. Int. Ed.* **2014**, *53* (39), 10398-10402.

(9) Corma, A.; Díaz-Cabañas, M. J.; Jiang, J.; Afeworki, M.; Dorset, D. L.; Soled, S. L.; Strohmaier, K. G. Extra-Large Pore Zeolite (ITQ-40) with the Lowest Framework Density Containing Double Four- and Double Three-Rings. *PNAS.* **2010**, *107* (32), 13997-14002.

(10) Pulido, A.; Sastre, G.; Corma, A. Computational Study of 19F NMR Spectra of Double Four Ring-Containing Si/Ge-Zeolites. *ChemPhysChem* **2006**, *7* (5), 1092-1099.

(11) Chen, C.-Y.; Finger, L. W.; Medrud, R. C.; Kibby, C. L.; Crozier, P. A.; Chan, I. Y.; Harris, T. V.; Beck, L. W.; Zones, S. I. Synthesis, Structure, and Physicochemical and Catalytic Characterization of the Novel High-Silica Large-Pore Zeolite SSZ-42. *Chem. Eur. J.* **1998**, *4* (7), 1312-1323.
